# Supplementary material for: Coordinated active repression operates via transcription factor cooperativity and multiple inactive promoter states in a developing organism
Source: Nat Commun. 2025 Sep 1;16:8157. doi: 10.1038/s41467-025-62907-3 (PMC12402238; doi:10.1038/s41467-025-62907-3)
Supplement: Supplementary file 1 — Supplementary Information [file 41467_2025_62907_MOESM1_ESM.pdf]

Coordinated active repression operates via transcription factor cooperativity and multiple inactive promoter states in a developing organism

Virginia Pimmett[1], Maria Douaihy[1,2,*], Louise Maillard[1,*], Antonio Trullo[1], Pablo Garcia Idieder[1], Melissa Costes[1], Jeremy Dufourt[1,3], Helene Lenden-Hasse[1], Ovidiu Radulescu[2#] and Mounia Lagha[1#]

Supplementary Information

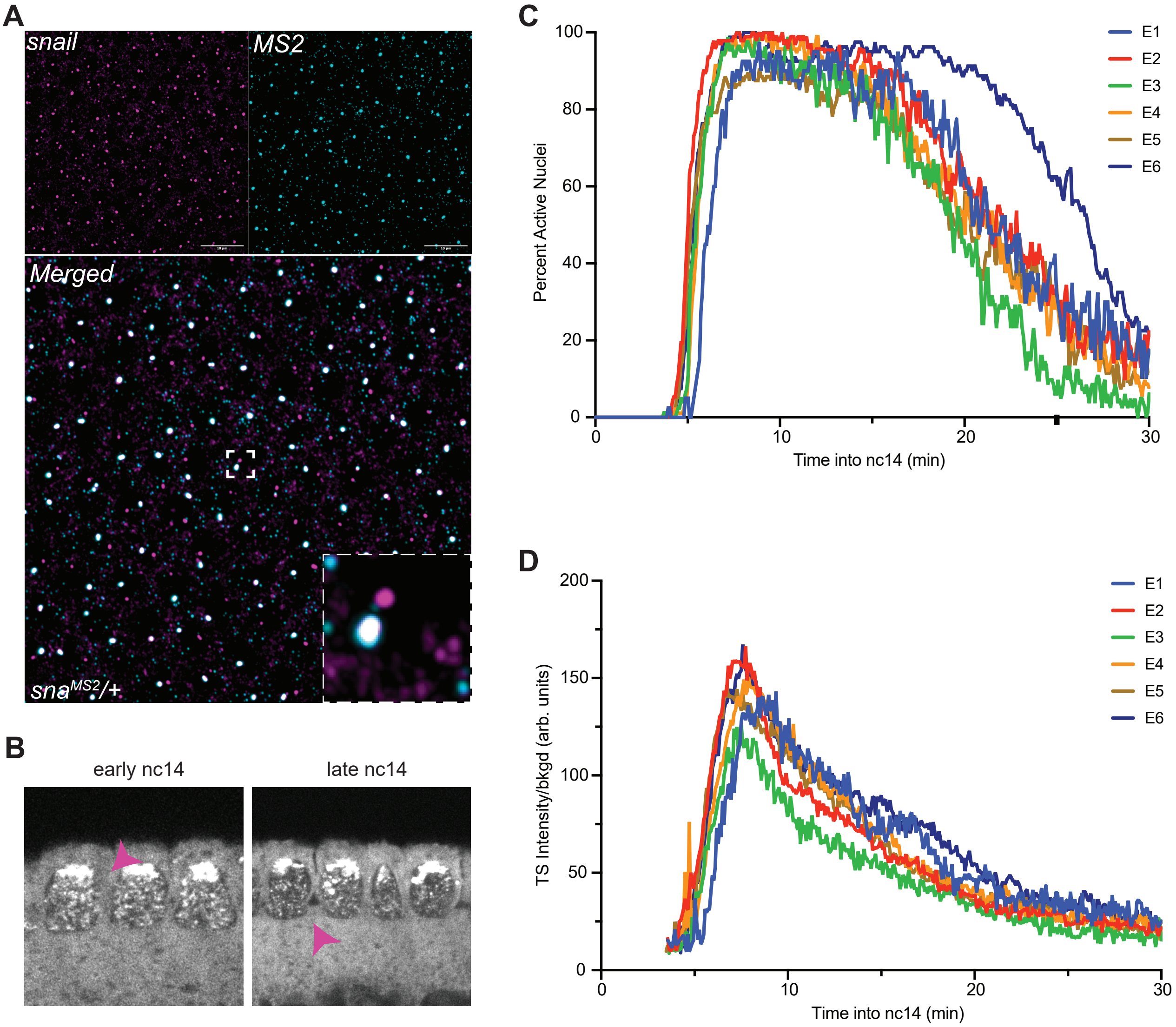

Supplementary Figure 1: Profiling *sna*^{MS2}. A) *sna*$^{MS2/+}$ heterozygotes showing coincident labelling of *sna*^{MS2} allele and endogenous snail mRNA at the transcription site (inset). Scale bar represents 10 mm. B) Sample membrane invagination (arrowheads) indicating partitioning of embryos into early (left) and late (right) nc14. C) Activation profiles for individual *sna*$^{MS2/+}$ movies during nc14. D) Average intensity of actively transcribing nuclei for individual *sna*$^{MS2/+}$ movies.

Statistics: *sna*$^{MS2/+}$ nuclear cycle 14: N=6 embryos, n=484 nuclei.

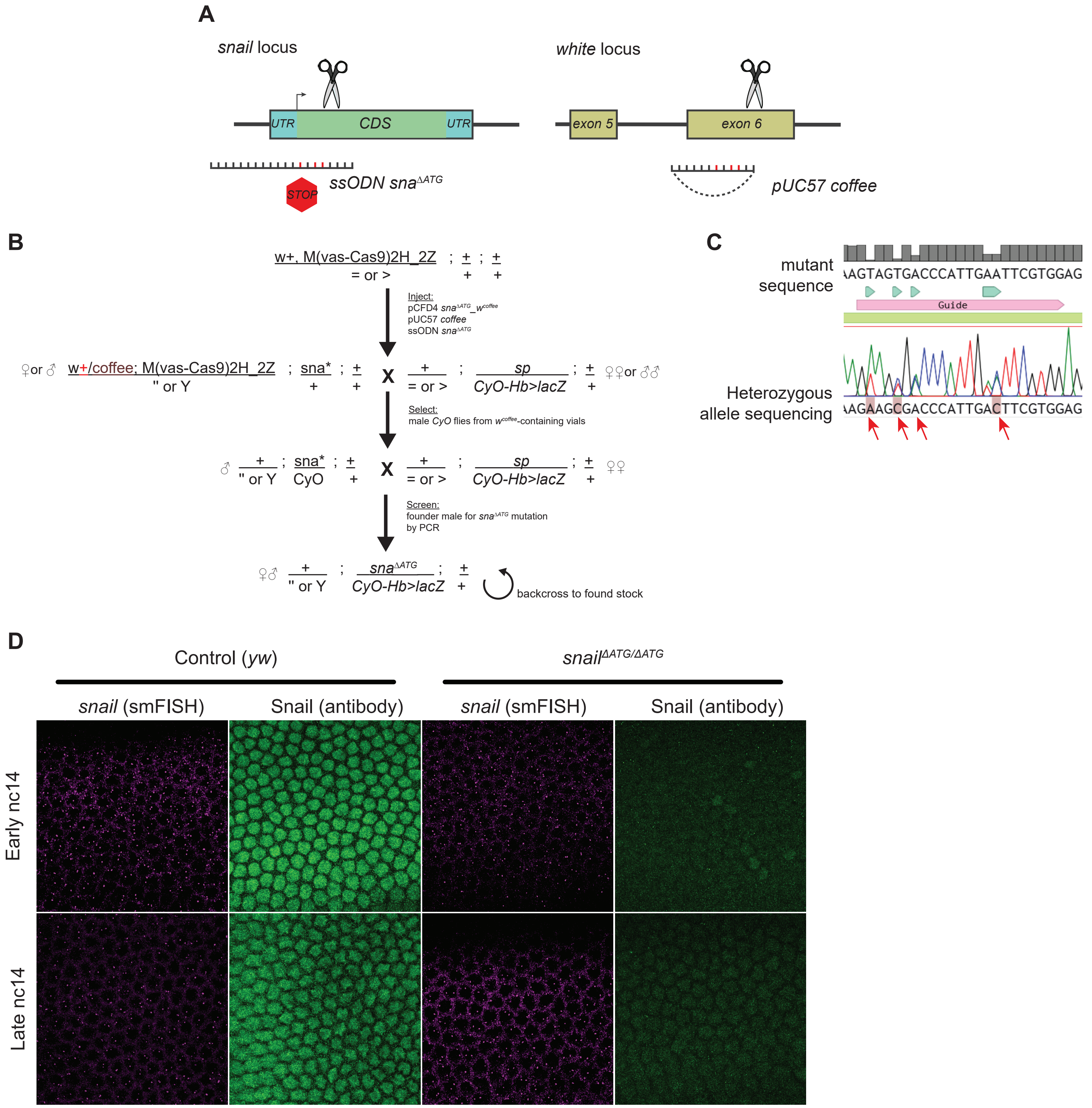

Supplementary Figure 2: Generation of *sna*$^{\Delta ATG}$*/CyO-Hb>lacZ* line. A) schematic of co-CRISPR strategy targeting *sna* and *w* simultaneously. B) Crossing scheme to recover mutant *sna*$^{\Delta ATG}$ alleles. Adapted from Levi et al. C) Sequencing results of fly. Arrows indicate mutations relative to wild type sequence. D) smFISH-IF to show co-occurrence of *sna* transcription with concomitant expression of Sna protein in *yw* control and absence of Sna protein in *sna*$^{\Delta ATG/\Delta ATG}$.

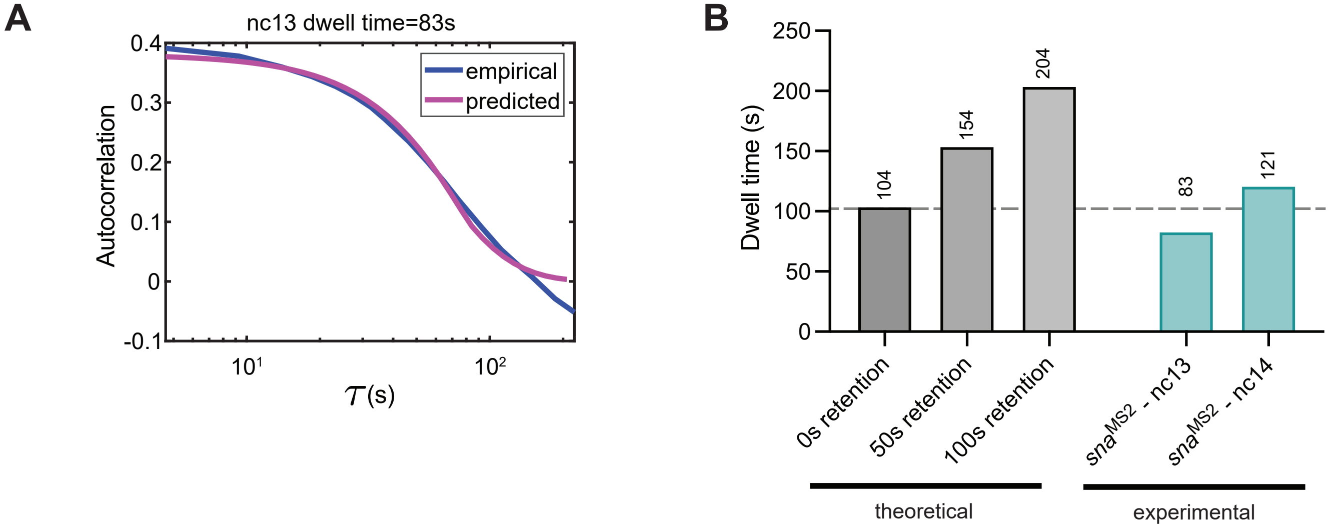

Supplementary Figure 3: Autocorrelation of the sna transcription signal in nuclear cycle 13. A) Sample trace demonstrating autocorrelation of a *sna* transcription trace in nc14. B) Dwell time of the sna^{MS2} signal in nuclear cycles 13 and 14 compared to the theoretical prediction for a polymerase speed of 25bp/s and a retention time at the transcription site of 0s.

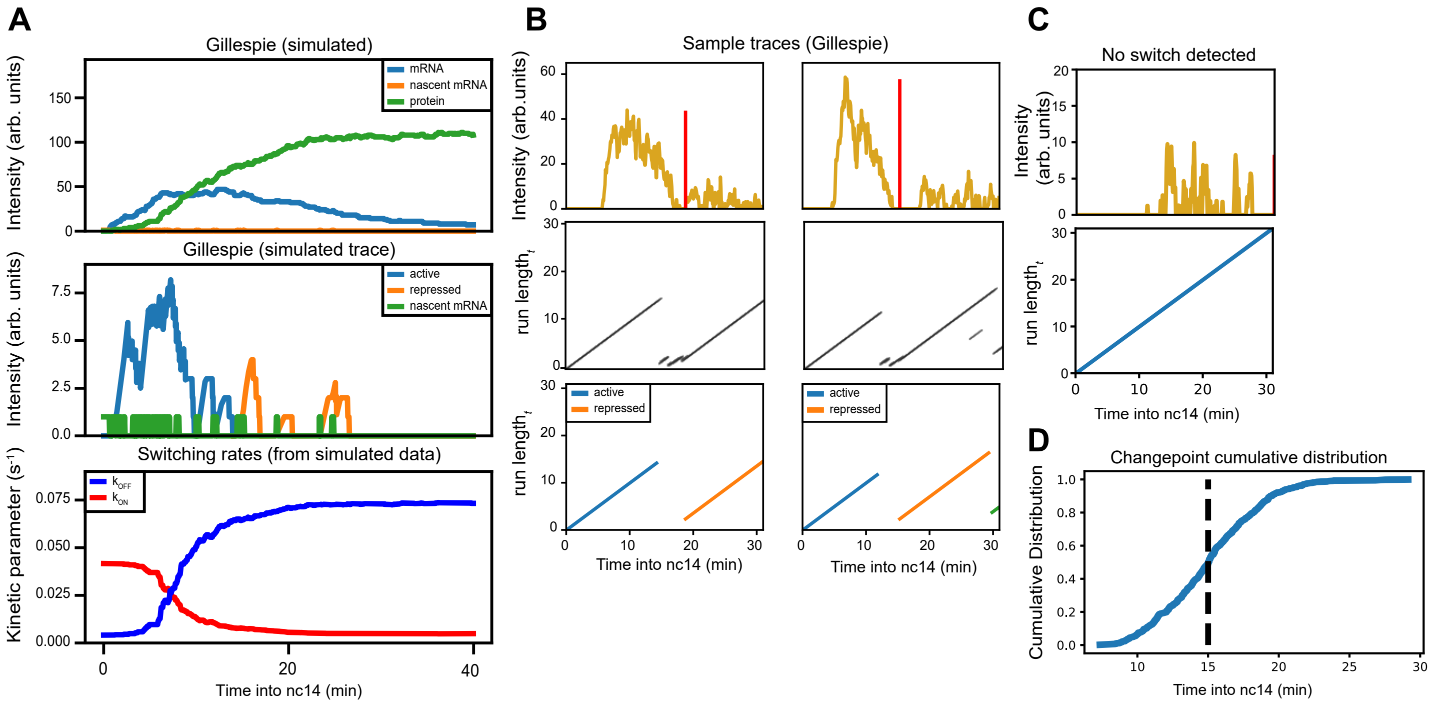

Supplementary Figure 4: Bayesian Change Point Detection (BCPD) identifies active and repressed periods. A) Synthetic data generated using the Gillespie algorithm with a two-state auto-repressive model. In this model, population-level k_{ON} and k_{OFF} depend on the protein level according to decreasing and increasing Hill functions, respectively (top). Synthetic traces were computed with a detected change point highlighted by a change in colour (middle) and a global change in kinetic parameters was observed (bottom). B) Synthetic traces generated using the Gillespie algorithm with their respective run length identified by the BCPD algorithm and classification into active and repressed states. C) Sample synthetic trace with no change in activation/repression is correctly detected by the BCPD algorithm. D) Cumulative distribution of BCPD algorithm-identified changepoints for synthetic data compared to the repression identified by the Hill algorithm.

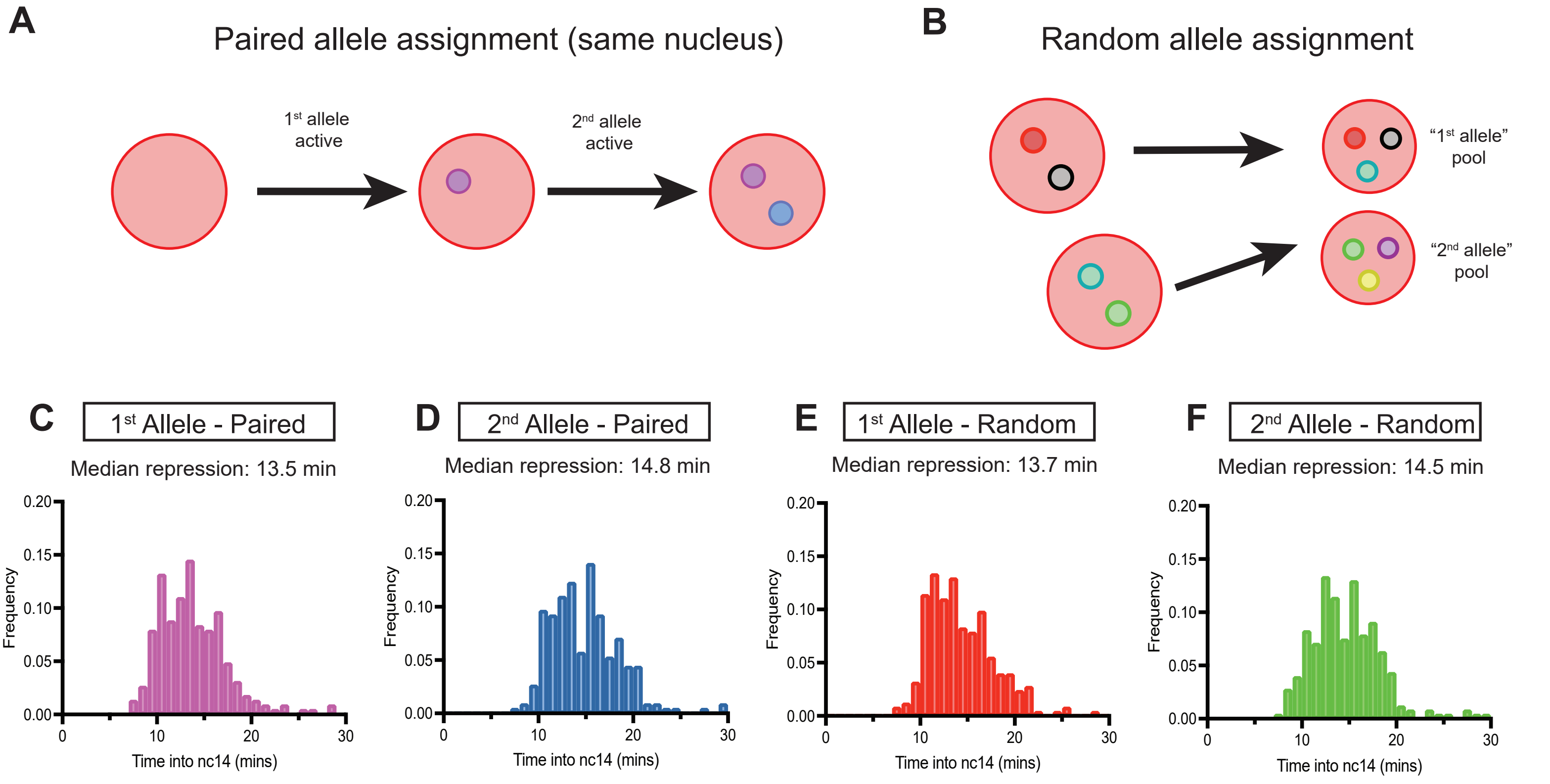

Supplementary Figure 5: Dual allele labelling of *sna*$^{MS2/MS2}$ shows no difference in repression onset between alleles. A) Schematic of 'paired' allele assignment dividing alleles in the same nucleus into separate analysis pools based on initial activation order. B) Schematic of 'random' allele assignment where alleles were randomly sorted into two equal sized pools irrespective of nucleus or activation order. C-D) Distribution of repression onset for first active allele (C) or second active allele (D). E-F) Distribution of repression onset for each of the randomly assigned pools.

Statistics: *sna*$^{MS2/MS2}$ paired alleles: N=3 embryos, n=228 nuclei per class; *sna*$^{MS2/MS2}$ randomly assigned alleles: N=3 embryos, n=255 nuclei ('first' allele) and n=256 nuclei ('second' allele).

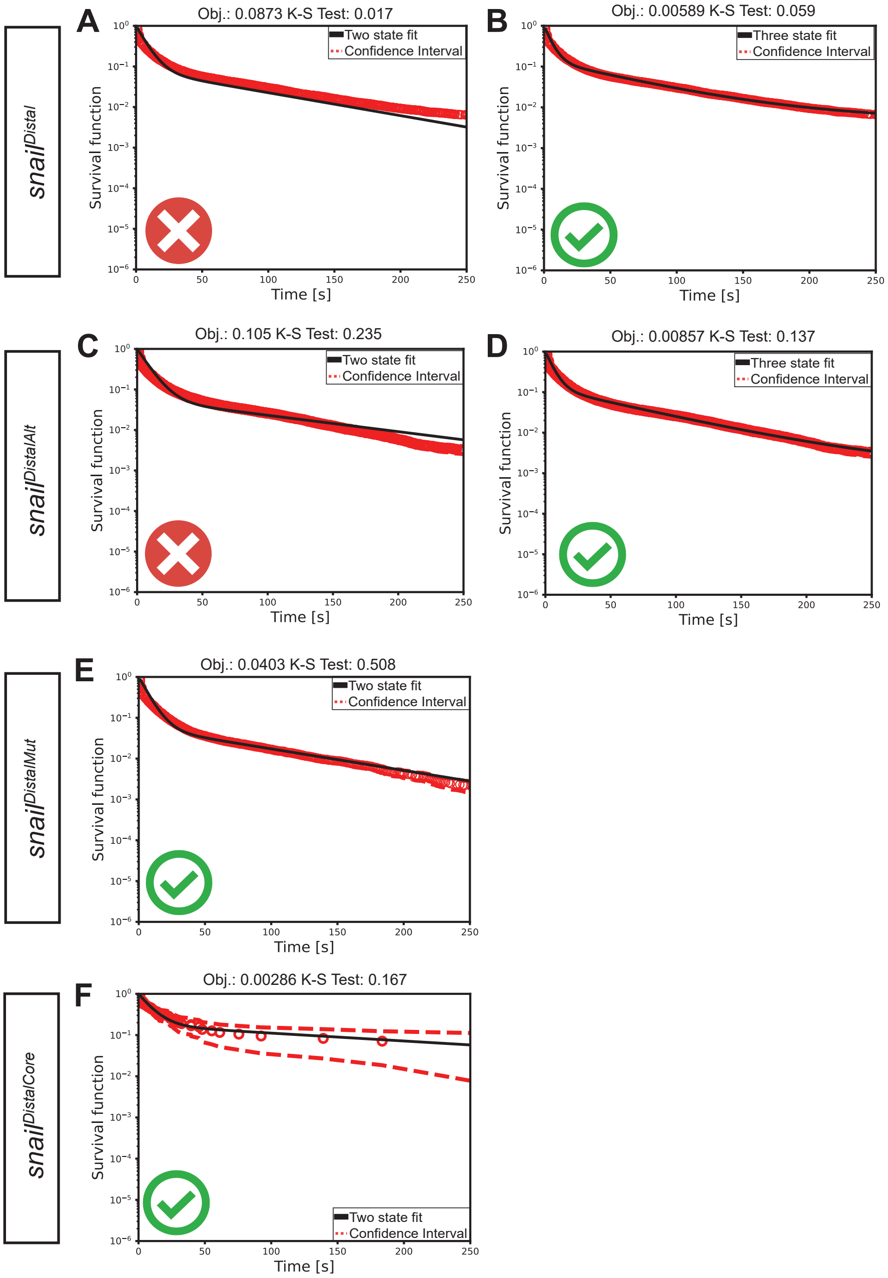

Supplementary Figure 6: Survival functions for *sna*^{Distal} transgene series in nc14. A-B) Survival function of the distribution of waiting times between polymerase initiation events (red circles) for *sna*^{Distal} with the two-exponential fitting (A) or three-exponential fitting (B) of the population estimated using the Kaplan–Meyer method (black line). The dashed lines indicate 95% confidence interval. A red cross indicates a rejected fitting. A green check indicates an accepted fitting. C-D) Survival function of the distribution of waiting times between polymerase initiation events (red circles) for *sna*^{DistalAlt} with the two-exponential fitting (C) or three-exponential fitting (D) of the population estimated using the Kaplan–Meyer method (black line). The dashed lines indicate 95% confidence interval. A red cross indicates a rejected fitting. A green check indicates an accepted fitting. E) Survival function of the distribution of waiting times between polymerase initiation events (red circles) for *sna*^{DistalMut} showing a two-exponential fitting of the population estimated using the Kaplan–Meyer method (black line). The dashed lines indicate 95% confidence interval. A green check indicates an accepted fitting. F) Survival function of the distribution of waiting times between polymerase initiation events (red circles) for *sna*^{DistalCore} showing a two-exponential fitting of the population estimated using the Kaplan–Meyer method (black line). The dashed lines indicate 95% confidence interval. A green check indicates an accepted fitting.

Statistics: *sna*^{Distal} N=3 embryos, n=224 nuclei; *sna*^{DistalAlt} N=5 embryos, n=220 nuclei; *sna*^{DistalMut} N=2 embryos, n=145 nuclei; *sna*^{DistalCore} N=3 embryos, N=194 nuclei.

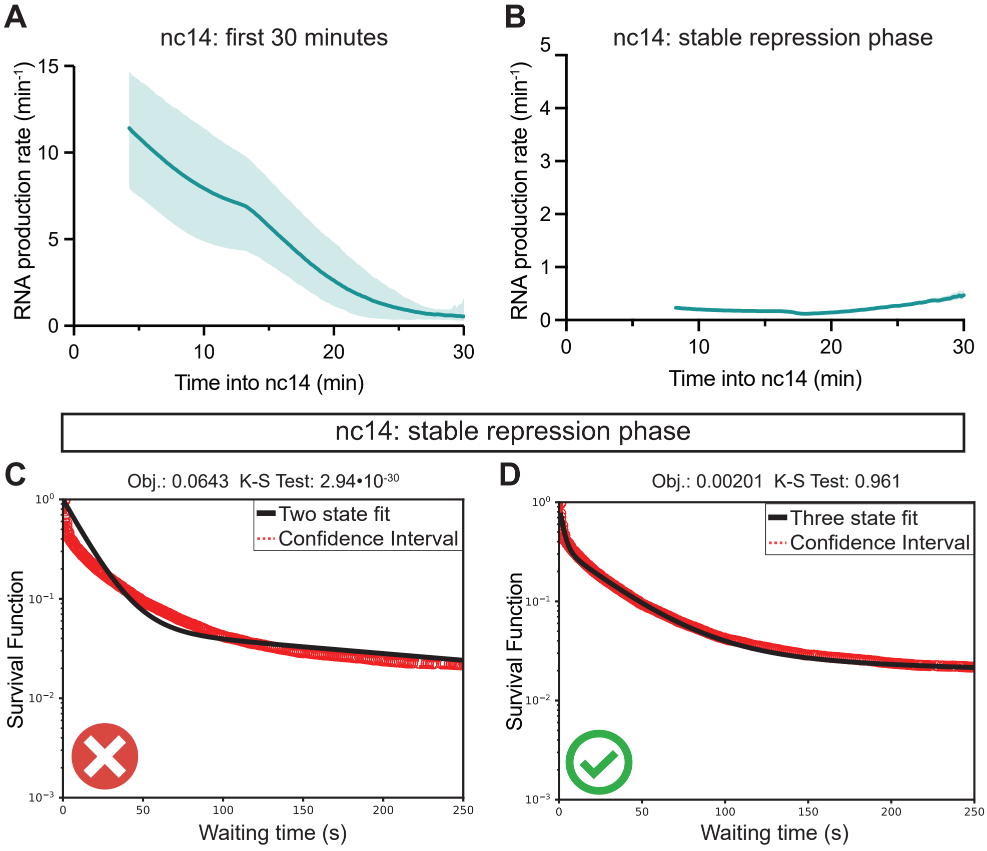

Supplementary Figure 7: *sna* expression in nc14 is stationary only during stable repression. A-B) Kinetic parameter stability as a function of time in nc14 during the first 30 minutes (A) or during stable repression only (B). Transcription expressed as the product of the probability to be active (pON) and the RNA polymerase II initiation rate (k_{ini}). C-D) Survival function of the distribution of waiting times between polymerase initiation events (red circles) for stable repression of sna with the two-exponential fitting (C) or three-exponential fitting (D) of the population estimated using the Kaplan–Meyer method (black line). The dashed lines indicate 95% confidence interval. A red cross indicates a rejected fitting. A green check indicates an accepted fitting.

Statistics: $sna^{MS2/+}$ nuclear cycle 14: N=6 embryos, n=484 nuclei.

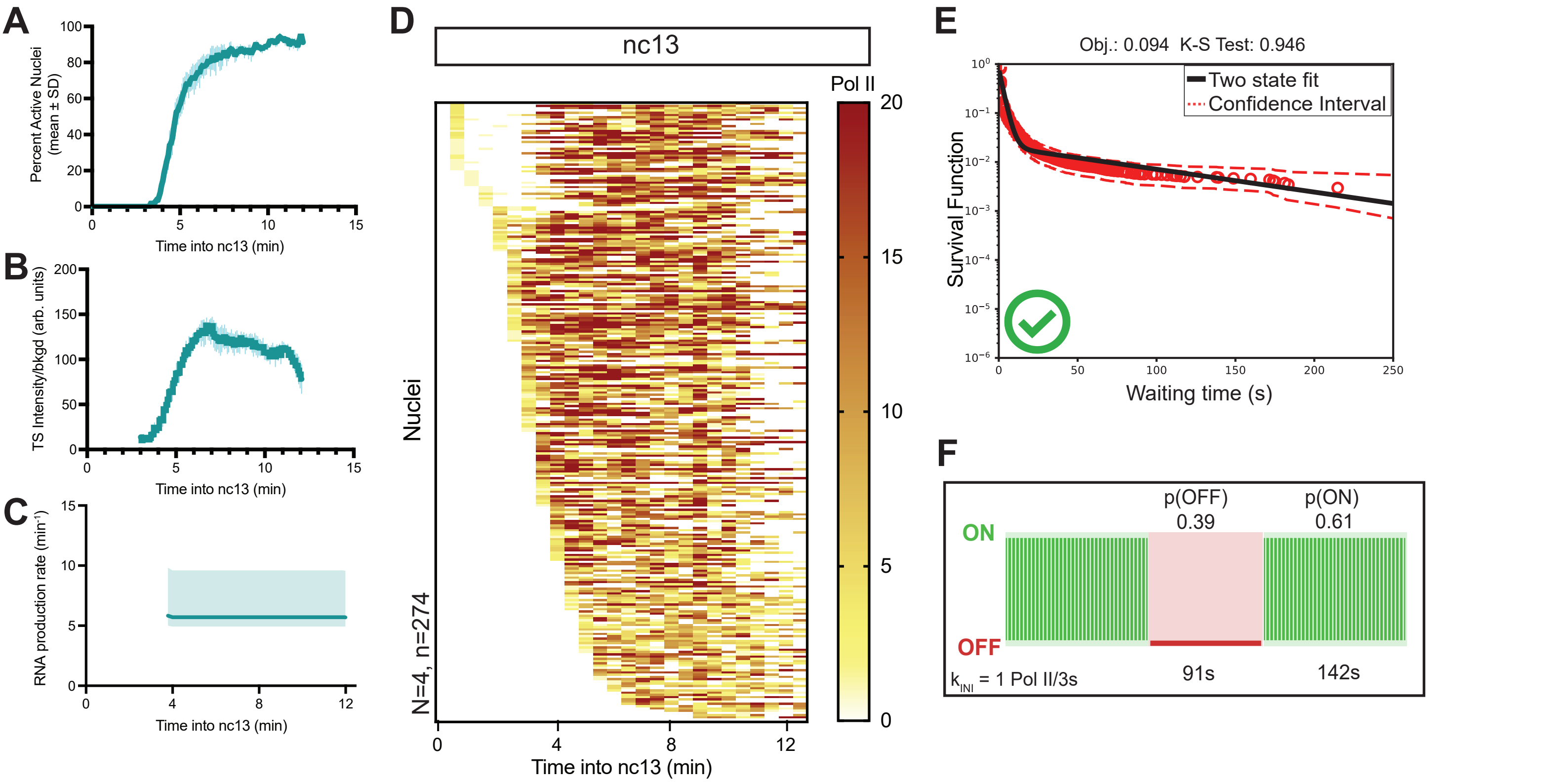

Supplementary Figure 8: *sna* expression in nc13 is in a fully active two-state regime. A) Instantaneous activation percentage (mean ± SEM) curves of ventral nuclei during nc13. Time zero is from anaphase during nc12-nc13 mitosis. B) Fluorescence intensity of actively transcribing nuclei (mean ± SEM) during nc13. Time zero is from anaphase during nc12-nc13 mitosis. C) Kinetic parameter stability as a function of time in nc13. Transcription expressed as the product of the probability to be active (pON) and the RNA polymerase II initiation rate (k_{ini}). D) Heatmap showing the number of polymerase initiation events in nc14 for *sna* in nc13 as a function of time. Each row represents one nucleus, and the number of Pol II initiation events per 30 s bin is indicated by the bin color. E) Survival function of the distribution of waiting times between polymerase initiation events (red circles) for sna showing a two-exponential fitting of the population estimated using the Kaplan–Meyer method (black line). The dashed lines indicate 95% confidence interval. A green check indicates an accepted fitting. F) Representation of estimated bursting dynamics for *sna* in nc13. Permissive ON state durations are depicted in green and inactive OFF states in red and orange, and probabilities of each state shown above.

Statistics: $sna^{MS2/+}$ nuclear cycle 13: N=4 embryos, n=274 nuclei.

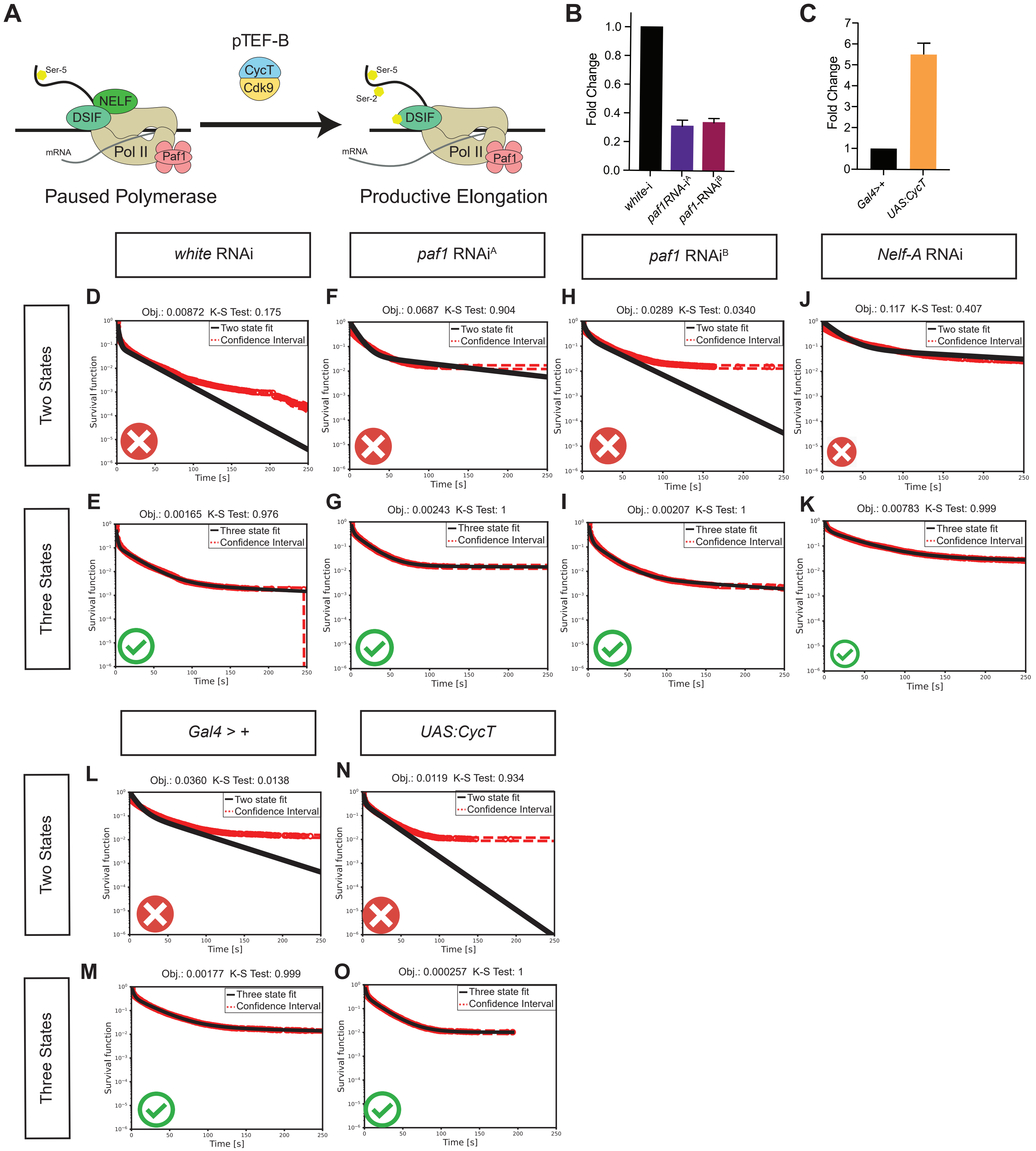

Supplementary Figure 9: Pausing is not rate-limiting during *sna* stable repression. A) schematic of pausing-related controls mediated by the Paf1 and pTEF-B complexes. B-C) Quantification of *paf1* knockdown (B) or *Cyclin T* over-expression (C) relative to indicated controls. D-K) Survival function of the distribution of waiting times between polymerase initiation events (red circles) for *white*-RNAi (D,E), *paf1*-RNAi^{A} (F,G) *paf1*-RNAi^{B} (H, I), and *Nelf-A* RNAi (J,K) with a two- (upper row) or three-exponential (lower row) fitting of the population estimated using the Kaplan–Meyer method (black line). L-O) Survival function of the distribution of waiting times between polymerase initiation events (red circles) for *Gal4*>+ (L,M) and *UAS:CycT* (N,O) with a two- (upper row) or three-exponential (lower row) fitting of the population estimated using the Kaplan–Meyer method (black line). Dashed lines indicate 95% confidence interval. A green check indicates an accepted fitting.

Statistics: *white*-RNAi > *sna*$^{MS2/+}$: N=2 embryos, n=183 nuclei; *paf1*-RNAi^{A} > *sna*$^{MS2/+}$: N=2 embryos, n=177 nuclei; *paf1*-RNAi^{B}> *sna*$^{MS2/+}$: N=2 embryos, n=184 nuclei; *Nelf-A* RNAi> *sna*$^{MS2/+}$: N=2 embryos, n=154 nuclei; *Gal4*>+> *sna*$^{MS2/+}$: N=2 embryos, n=191 nuclei; *UAS:CycT*> *sna*$^{MS2/+}$: N=2 embryos, n=154 nuclei.

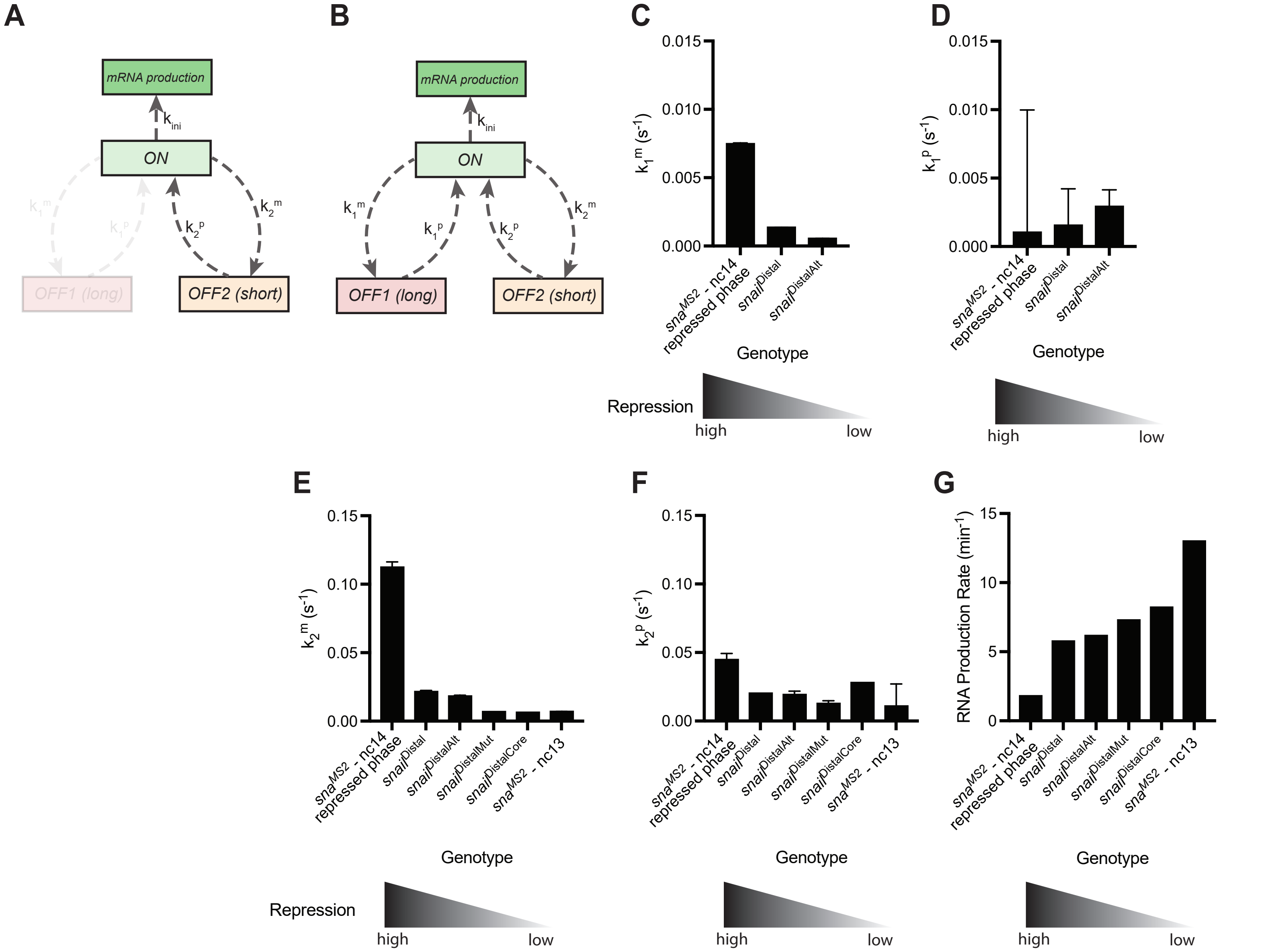

Supplementary Figure 10: Comparison of kinetic parameters for endogenous *sna* and sna^{Distal} transgene mutant series. A-B) Schema of two state (A) and three state (B) topology with transitions indicated. For two-state genotypes, the k1m and k1p transitions cannot be reached. C-G) Comparison of state transition rates for k1m (C), k1p (D), k2m (E), k2p (F), and the RNA production rate (G). Genotypes are arranged in order of decreasing repression.

A **B**

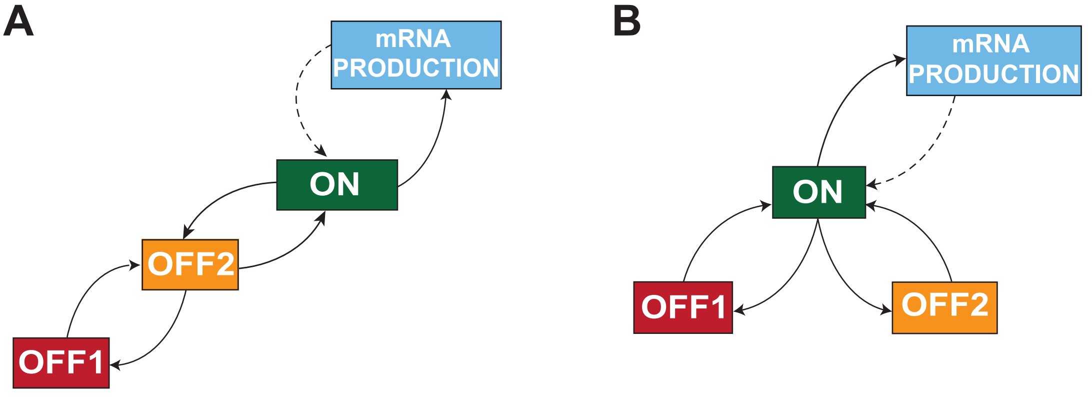

Sequential Model

Non-sequential Model

C

***snail*^{Distal}**

	p(ON)	p(OFF1)	p(OFF2)	T(ON) (s)	T(OFF1) (Long) (s)	T(OFF2) (Short) (s)	k_{INI} (s/initiation event)
Sequential Model	0.34	0.25	0.41	43.5	605	49.4	6
Non-sequential Model	0.34	0.30	0.36	43.5	640	49.1	6

***snail*^{DistalAlt}**

	p(ON)	p(OFF1)	p(OFF2)	T(ON) (s)	T(OFF1) (s)	T(OFF2) (s)	k_{INI} (s/initiation event)
Sequential Model	0.53	0.07	0.40	61.7	265.7	44.6	6
Non-sequential Model	0.53	0.10	0.37	61.7	275.3	44.3	6

Supplementary Figure 11: Comparison of sequential and non-sequential three-state kinetic topologies for *snail*^{Distal} and *snail*^{DistalAlt} transgenes. A-B) schemas representing the topologies of the sequential (A) and non-sequential (B) three-state kinetic topologies. C) Comparison of kinetic parameters for indicated genotypes derived from fitting to sequential or non-sequential topologies respectively.

Supplementary Data 1: Kinetic parameters for indicated genotypes

Genotype	2 States Model									
	**k2+**	**k2-**	**k3**	**T(OFF) (s)**	**T(On) (s)**	**Pol II Initiaiton (s/event)**	**p(OFF)**	**p(ON)**	**Objective function**	**Kolmogorov-Smirnov**
***sna* MS2 - active phase (nc13)**	**0.011**	**0.007**	**0.350**	**91**	**142**	**2.85**	**0.392**	**0.608**	**0.095432177**	**0.985401194**
Minimum	0.011	0.005	0.330	91	192	3.03	0.161	0.608		
Maximum	0.027	0.007	0.350	37	142	2.85	0.392	0.839		

Genotype	3 States Model													
	**k1+**	**k1-**	**k2+**	**k2-**	**k3**	**T(OFF1) (s)**	**T(OFF2) (s)**	**T(On) (s)**	**Pol II Initiaiton (s/event)**	**p(OFF1)**	**p(OFF2)**	**p(ON)**	**Objective function**	**Kolmogorov-Smirnov**
**snaMS2 - repressed phase (nc14)**	**0.001**	**0.007**	**0.045**	**0.112**	**0.271**	**956**	**22**	**8**	**3.68**	**0.671**	**0.236**	**0.094**	**0.002011144**	**0.961125388**
Minimum	0.001	0.007	0.045	0.112	0.271	956	22	8	3.68	0.184	0.236	0.094		
Maximum	0.010	0.008	0.049	0.116	0.284	100	20	8	3.52	0.671	0.573	0.243		

Genotype	3 States Model													
	**k1+**	**k1-**	**k2+**	**k2-**	**k3**	**T(OFF1) (s)**	**T(OFF2) (s)**	**T(On) (s)**	**Pol II Initiaiton (s/event)**	**p(OFF1)**	**p(OFF2)**	**p(ON)**	**Objective function**	**Kolmogorov-Smirnov**
snailDistal	**0.002**	**0.001**	**0.020**	**0.022**	**0.171**	**640**	**49**	**44**	**5.84**	**0.299**	**0.361**	**0.340**	**0.005894326**	**0.059164976**
Minimum	0.002	0.000	0.020	0.022	0.171	237	49	44	5.75	0.029	0.361	0.340		
Maximum	0.004	0.001	0.020	0.022	0.174	640	49	44	5.84	0.299	0.510	0.462		
snailDistalAlt	**0.003**	**0.001**	**0.019**	**0.018**	**0.160**	**342**	**52**	**53**	**6.23**	**0.089**	**0.443**	**0.468**	**0.008572441**	**0.137155036**
Minimum	0.003	0.000	0.019	0.018	0.160	241	46	52	6.08	0.042	0.443	0.468		
Maximum	0.004	0.001	0.022	0.019	0.165	342	52	53	6.23	0.089	0.446	0.512		

Genotype	2 States Model									
	**k2+**	**k2-**	**k3**	**T(OFF) (s)**	**T(On) (s)**	**Pol II Initiaiton (s/event)**	**p(OFF)**	**p(ON)**	**Objective function**	**Kolmogorov-Smirnov**
snailDistalMut	**0.013**	**0.007**	**0.135**	**78**	**145**	**7.40**	**0.351**	**0.649**	**0.040272118**	**0.507765922**
Minimum	0.013	0.006	0.134	68	145	7.40	0.298	0.649		
Maximum	0.015	0.007	0.135	78	159	7.45	0.351	0.702		
snailDistalCore	**0.028**	**0.006**	**0.124**	**36**	**154**	**8.10**	**0.188**	**0.812**	**0.028555408**	**0.1666541**
Minimum	0.028	0.006	0.124	36	154	8.10	0.188	0.812		
Maximum	0.028	0.006	0.124	36	154	8.10	0.188	0.812		

Supplementary Data 2: Fly lines associated with Pimmett et al. (2025)

Line	In Text Reference	Reference
; ; nos> MCP-eGFP, His2A-mRFP		Gift from T.Fukaya
; *Mat-alpha* :GAL4/CyO; *nos* :GAL4, *nos* >MCP-eGFP, His2A-RFP/*nos* :GAL4, nos>MCP-eGFP, His2A-RFP		Lagha lab
; *snailMS2-3xP3-dsRed/snailMS2-3xP3-dsRed ;*	*snaMS2*	This paper
; *sna ΔATG /* CyO, *hb* >lacZ ;	*snaΔATG*	This paper
sogMS2/sogMS2 ; ;	*sogMS2*	Whitney et al., Development 2022
; *snailLlama/snailLlama* ;	*SnailLlama*	This paper
yw; P{w[+mC] = EGFP-STOP-bcd} ;	*bcd>* GFP	Bothma et al., Cell 2018
w ; *P{w[+mC]=His2Av-mRFP}/ CyO ;*	His2A-RFP	
w1118 ; *P{GD9782}v20876* ;	Paf1 RNAi-A	VDRC 20876
; ; *P{KK100080}VIE-260B*	Paf1 RNAi-B	VDRC 108826
; ; *P{UASp-CycT.H}*	UAS:CycT	Hunt et al., Genome Biology 2024
; ; *P{y[+t7.7] v[+t1.8]=TRiP.HMS00686}attP2*	Nelf-A RNAi	BDSC 32897
; *PBac{sna-MS2-y}* ;	*snaWT* BAC	Bothma et al., eLife 2015
; *PBac{snaΔprimary-MS2-y}* ;	*snaΔPROX* BAC	Bothma et al., eLife 2015
; *PBac{snaΔshadow-MS2-y}* ;	*snaΔDIST* BAC	Bothma et al., eLife 2015
; ; PBPhi(*snaDistal-24xMS2-y)* (VK33)	*snaDistal*	Dufourt et al., Nature Communications 2018
; ; PBPhi(*snaDistalAlt-24xMS2-y)* (VK33)	*snaDistalAlt*	This paper
; ; PBPhi(*snaDistalMut-24xMS2-y)* (VK33)	*snaDistalMut*	This paper
; ; PBPhi(*snaDistalCore-24xMS2-y)* (VK33)	*snaDistalCore*	Ferraro et al., Current Biology 2016

Supplementary Data 3: guide RNA sequences for generation of CRISPR alleles

Target	Sequence
snail – MS2	CGACATATGAATCCCTTAGCAGG
snail – Llama	CGACATATGAATCCCTTAGCAGG, CCCCATGAACGAAGAGTACTAGG
snail – ΔATG (guide)	GTAGTGACCCATTGAATTCGTGG
snail – ΔATG (ssODN)<br>**guide sequence**<br>mutations<br>EcoRI site for screening	TCGATCAGTACCGGAAACTAAAACTTAATCACACACACATCAAAAATGGCCGCC AACTACAAAAGCTGCCCGCTAAA**GTAGTGACCCATTGAATTCGTGG**AGGAGC GTCTGCCACAAACGGAGGCCTTGGCCCTGACCAAGGACTCACAGTTTGCCCA GGATCAGCCGCAGGATCTATCCCTGAAACGGGGTCGCGACGA
White - coffee	ATACCATTCCTGCTCTTTGG

Supplementary Data 4: *snail* Distal Enhancer sequences

Genotype	Sequence
snailDistal (WT)	cgcattgaggtgttttgttggtcaacgccaaacatgtgcttgccacatgccccgtttcggacccattttcaataccaaactcaagcctgtccaggcgaaggtgtgaactatttggcctggtgccttatctacacttagaaaaatattgacataaaatggatttttgaacggtgttaaacaaaacggtgtttgttttaaacaaaatacttcaaaattcgtaaaatacgattatcgttaaaacccatcgtaaccatttaaatataccctttcataccatttacatacttaattatttaaaatagaagaggttcaacatatattttccgttttccaaactagttgacatttttttgcggtgtaactgtgatagctgtgtaccttcaaagtcccatgtgccagtctctctcaccttggtcctaccttcgacttcgctgggctctccgttttcccatgagggagttctcctggcccgctcacatatgtcggaactccaactgcggagctgggaaaacaaacaggactggcggtactggttacacatgtgtgagctggccggaccggagcacgtactatgcgcgtaggtgcgactccttggattccccagttctccagttcccacacatcctggccgggccacatgatgatgttgctgtgcttttcccgtagcgccaggcgtgtccaaatgttttgttgcggaattcctcaaggcgaggaatctcgcacacattacgagtagtcggcgctcgcggaaaaaaaacacacgagccaaagttacatatgttctttgggccatttattcaagaaatttccatgtcggccgaaaagggatttccttgtaggcggcgttttcccacgattttataactgggctcgacttcccatcttcccaccgtccatgtgttgctgggaaatcggcgttgcctttggtttttttgccttgcgggggaaaagttctgatttgagtgcgctcgagactttccagctaggagcaggacatgtggccggtagccagtgccactcagtacctcactcagtctaatggccagaacaccgcggtcatctcgagtggacaggttgtaaagtggccacacccgccgaccggacacttgactctatgcgccggcgcagcatcgcaggtagtcatctggaacaggtaaacatttgccaatatctcacgctgacctatgacacgtgtcaggtgtctttgtcgtggattcgggcaagtgtgaaacaacctactacctgttgaggcaataatgattatgaatacgaatgaattaacacggagaacgataagagggccaattgcatttgttccttcttccaaaacgagctgcaaacggcagctaattgtattttaataattataaatccgtgttaaattgtttgaaatttaataaattggttcctttgcaaaaacagcttaaatggaaatactgcaattttgtccatattttattgtcatcgaattttcaaatgg
snailDistalAlt	cgcattgaggtgttttgttggtcaacgccaaacatgtgcttgccacatgccccgtttcggacccattttcaataccaaactcaagcctgtccaggcgaaggtgtgaactatttggcctggtgccttatctacacttagaaaaatattgacataaaatggatttttgaacggtgttaaacaaaacggtgtttgttttaaacaaaatacttcaaaattcgtaaaatacgattatcgttaaaacccatcgtaaccatttaaatataccctttcataccatttacatacttaattatttaaaatagaagaggttcaacatatattttccgttttccaaactagttgacatttttttgcggtgtaactgtgatagctgtgtaccttcaaagtcccatgtgccagtctctctcaccttggtcctaccttcgacttcgctgggctctccgttttcccatgagggagttctcctggcccgctcacatatgtcggaactccaactgcggagctgggaaaacaaacaggactggcggtactggttacacatgtgtgagctggccggaccggagcacgtactatgcgcgtaggtgcgactccttggattccccagttctccagttcccacacatcctggccgggccacatgatgatgttgctgtgcttttcccgtagcgccaggcgtgtccaaatgttttgttgcggaattcctcaaggcgaggaatctcgcacacattacgagtagtcggcgctcgcggaaaaaaaacacacgagccaaagttacatatgttctttgggccatttattcaagaaatttccatgtcggccgaaaagggatttccttgtaggcggcgttttcccacgattttataactgggctcgacttcccatcttcccaccgtccatgtgttgctgggaaatcggcgttgcctttggtttttttgccttgcgggggaaaagttctgatttgagtgcgctcgagactttccagctaggagcagcgtaagtgcccggtagccagtgccactcagtacctcactcagtctaatggccagaacaccgcggtcatctcgagtggacaggttgtaaagtggcccgtaatagtgaccggacacttgactctatgcgccggcgcagcatcgcaggtagtcatctgcgtaatagtcgtatttgccaatatctcacgctgacctatgacacgtgtcaggtgtctttgtcgtggattcgcgtaagtgcgaaacaacctactacctgttgaggcaataatgattatgaatacgaatgaattaacacggagaacgataagagggccaagcgtaatagtcgtttcttccaaaacgagctgcaaacggcagctaattgtattttaataattataaatccgtgttaaattgtttgaaatttaataaattggttcctttgcaaaaacagcttaaatggaaatactgcaattttgtccatattttattgtcatcgaattttcaaatgg
snailDistalMut	cgcattgaggtgttttgttggtcaacgccaaacatgtgcttgccacatgccccgtttcggacccattttcaataccaaactcaagcctgtccaggcgaaggtgtgaactatttggcctggtgccttatctacacttagaaaaatattgacataaaatggatttttgaacggtgttaaacaaaacggtgtttgttttaaacaaaatacttcaaaattcgtaaaatacgattatcgttaaaacccatcgtaaccatttaaatataccctttcataccatttacatacttaattatttaaaatagaagaggttcaacatatattttccgttttccaaactagttgacatttttttgcggtgtaactgtgatagctgtgtaccttcaaagtcccatgtgccagtctctctcaccttggtcctaccttcgacttcgctgggctctccgttttcccatgagggagttctcctggcccgctcacatatgtcggaactccaactgcggagctgggaaaacaaacaggactggcggtactggttacacatgtgtgagctggccggaccggagcacgtactatgcgcgtaggtgcgactccttggattccccagttctccagttcccacacatcctggccgggccacatgatgatgttgctgtgcttttcccgtagcgccaggcgtgtccaaatgttttgttgcggaattcctcaaggcgaggaatctcgcacacattacgagtagtcggcgctcgcggaaaaaaaacacacgagccaaagttacatatgttctttgggccatttattcaagaaatttccatgtcggccgaaaagggatttccttgtaggcggcgttttcccacgattttataactgggctcgacttcccatcttcccaccgtccatgtgttgctgggaaatcggcgttgcctttggtttttttgccttgcgggggaaaagttctgatttgagtgcgctcgagactttccagctaggagcagcgtaagtgcccggtagccagtgccactcagtacctcactcagtctaatggccagaacaccgcggtcatctcgagtggacaggttgtaaagtggcccgtaatagtgaccggacacttgactctatgcgccggcgcagcatcgcaggtagtcatctgcgtaatagtcgtatttgccaatatctcacgctgacctatgacacgtgtcaggtgtctttgtcgtggattcgcgtaagtgcgaaacaacctactacctgttgaggcaataatgattatgaatacgaatgaattaacacggagaacgataagagggccaagcgtaatagtcgtttcttccaaaacgagctgcaaacggcagctaattgtattttaataattataaatccgtgttaaattgtttgaaatttaataaattggttcctttgcaaaaacagcttaaatggaaatactgcaattttgtccatattttattgtcatcgaattttcaaatgg
snailDistalCore (Pimmett, Dejean et al., 2021)	ccttggtcctaccttcgacttcgctgggctctccgttttcccatgagggagttctcctggcccgctcacatatgtcggaactccaactgcggagctgggaaaacaaacaggactggcggtactggttacacatgtgtgagctggccggaccggagcacgtactatgcgcgtaggtgcgactccttggattccccagttctccagttcccacacatcctggccgggccacatgatgatgttgctgtgcttttcccgtagcgccaggcgtgtccaaatgttttgttgcggaattcctcaaggcgaggaatctcgcacacattacgagtagtcggcgctcgcggaaaaaaaacacacgagccaaagttacatatgttctttgggccatttattcaagaaatttccatgtcggccgaaaagggatttccttgtaggcggcgttttcccacgattttataactgggctcgacttcccatcttcccaccgtccatgtgttgctgggaaatcggcgttgcctttgg

Supplementary Data 5: single molecule FISH probes

snail endogenous smFISH probes

Probe	Sequence	Probe Type
snail_1	tctcaacgagagctgaggtg	smFISH
snail_2	gagtatagagcggtggttgc	smFISH
snail_3	tgggtaaatcgggagatcgg	smFISH
snail_4	agttttagtttccggtactg	smFISH
snail_5	ccatttttgatgtgtgtgtg	smFISH
snail_6	ttagcgggcagcttttgtag	smFISH
snail_7	ctcctccacgaagacaatgg	smFISH
snail_8	caaactgtgagtccttggtc	smFISH
snail_9	cgtttcagggatagatcctg	smFISH
snail_10	tgctgataatcctgggtctc	smFISH
snail_11	acatagtcacgtttcggttc	smFISH
snail_12	ccggtgtttttgaaaggttc	smFISH
snail_13	agttggagctagagctggag	smFISH
snail_14	tagtccacgcatatggattt	smFISH
snail_15	gattaatcgtggtgggggtg	smFISH
snail_16	atcacaaaggcggactggaa	smFISH
snail_17	cagagatcggattgcaaccg	smFISH
snail_18	atctgctggtagctgtagac	smFISH
snail_19	aaccggtttccagatcggat	smFISH
snail_20	actgaaagatcctctggctc	smFISH
snail_21	cggcagtgggatgtcatttc	smFISH
snail_22	gcctcatcgaaaaggtggaa	smFISH
snail_23	tgtaggagtatcccgatgag	smFISH
snail_24	catgattggcggcaacactc	smFISH
snail_25	gcacttgaagcggtagtttt	smFISH
snail_26	atcgaggtggagtacatctt	smFISH
snail_27	aactgacggtgcttggacag	smFISH
snail_28	ttcttctcctgattacactc	smFISH
snail_29	aatggtggtgtacagctttc	smFISH
snail_30	gtgcggatgtgcatcttcag	smFISH
snail_31	caaatggggcacttgcaggg	smFISH
snail_32	agggtcgagagaaggccttg	smFISH
snail_33	aaaggcttctctccagtgtg	smFISH
snail_34	caaaggatcgtgggcagtcg	smFISH
snail_35	gatgagctcgcaggttcgag	smFISH
snail_36	tacttcttgacgtccacgtg	smFISH
snail_37	gaaagatttgtggcacacct	smFISH
snail_38	tgcttgttcaggagcgacat	smFISH
snail_39	tagtgatggtgcagttggag	smFISH
snail_40	atatgtcgagaatcctacgc	smFISH
snail_41	taattgtgtcctgctaaggg	smFISH
snail_42	gcggaatgtgagtttgctta	smFISH
snail_43	attgtctgtttgtttggtct	smFISH
snail_44	gcaccaaaaccgaatcgact	smFISH
snail_45	atgctgcgtgtgacaatgag	smFISH
snail_46	acagttggcttaacagtact	smFISH
snail_47	ttcttctctttaagctagga	smFISH

MS2 smiFISH probes

Probe	Sequence	Probe Type
MS2_1	GATCGTCGTCGTTTGAAGATTCGACCTGG	smiFISH
MS2_2	CGGCTGATGCTCGTGCTTTCTTGGCA	smiFISH
MS2_3	CGTAGGATCTGATGAACCCTGGAATACTGG[illegible]	smiFISH

yellow smFISH probes

Probe	Sequence	Probe Type
yellow_1	atcagggtcacaagaatcca	smFISH
yellow_2	actatatcgctcctgaagtt	smFISH
yellow_3	tttagtcgggtattcgggaa	smFISH
yellow_4	tataatctccactagccaga	smFISH
yellow_5	ttcgactccaacaggtagag	smFISH
yellow_6	gtgacgaataaccgattgcc	smFISH
yellow_7	aaactgcggtccatgtttat	smFISH
yellow_8	gccaatctggatacggaatt	smFISH
yellow_9	caatctccagctgtatttga	smFISH
yellow_10	gtaggcagtggtaatactgt	smFISH
yellow_11	ccacactcatccactttaat	smFISH
yellow_12	cacggattagtggtggtatt	smFISH
yellow_13	gtatccgtggtcaagtcaaa	smFISH
yellow_14	tagctcgtatctccgaattc	smFISH
yellow_15	gtatttggatttgtgtccac	smFISH
yellow_16	cacggcaatgttagctatga	smFISH
yellow_17	atcatcgcaatttttgccta	smFISH
yellow_18	tatcccaattcatcggcaaa	smFISH
yellow_19	cccaggagtaagcaatcaag	smFISH
yellow_20	agaatctccaggacttgttc	smFISH
yellow_21	cctcaatggatcggggaaaa	smFISH
yellow_22	cccattggaagttaatacca	smFISH
yellow_23	ataccaaatataccctcctc	smFISH
yellow_24	cgatcgaatgggcgaaaggg	smFISH
yellow_25	agtacagggtacgataacca	smFISH
yellow_26	cgatgacttgctaacggact	smFISH
yellow_27	aaaatcctcgtggatacggc	smFISH
yellow_28	catgatagctatcttccgtc	smFISH
yellow_29	ccgttcatctaaggcaacaa	smFISH
yellow_30	cacgtgaagtggtatgggag	smFISH
yellow_31	acagctcaattccatcatcg	smFISH
yellow_32	gagtacggcattgatgagtg	smFISH
yellow_33	ccacaatgccatgaaattgc	smFISH
yellow_34	ttttcacatcggccggaaaa	smFISH
yellow_35	acccaaacgtttttgttctc	smFISH
yellow_36	gcaagaaaacgggcatccta	smFISH
yellow_37	aagggagccgtgtaaattcg	smFISH
yellow_38	aggcgttattcctcaaatca	smFISH
yellow_39	acggcttgttttggtattga	smFISH
yellow_40	atataacggtggacccattg	smFISH
yellow_41	tttctgtggcaagacaggac	smFISH
yellow_42	cgggcaaataagtgcgactt	smFISH
yellow_43	tggagactacattgcctgaa	smFISH
yellow_44	ggacccacagaatttgtaga	smFISH
yellow_45	ccgttgtgctggttgaaaat	smFISH
yellow_46	gaccacttgtctcgtaattt	smFISH
yellow_47	gggttgatgggtgggaaata	smFISH
yellow_48	aaccttgatgctgatgatgc	smFISH

Supplementary Data 6: qPCR Primers

Name	Sequence
paf1_F	CACCGCTTCGTGCAGTACAA
paf1_R	CCAAATCGTGTTCCGTCAGC
CycT_F	CCGGCCCGTCTGAAGTCTA
CycT_R	CCTTGCTGTTAGCTGTCCGAT
Rpl13_F	AGCGGCATGTGAAGACCTG
Rpl13_R	AAGACGGCCTTAGCCTTCTTG

Supplementary Data 1: Kinetic parameters for promoters derived from deconvolution and multi-exponential regression fitting of live imaging data. Minimum and maximum values indicate the boundaries of the error interval. State durations are calculated from the provided switching rates ($k_i^{+/-}$) and time durations for each state are provided as '*T(state)*'. State probability values are indicated as '*p(state)*'. Bold indicates the most parsimonious appropriate fitting of the data. The table also provides the objective functions and one-sided Kolmogorov-Smirnov test results.

Supplementary Data 2: *Drosophila* lines used in this manuscript.

Supplementary Data 3: guide RNA and ssODN sequences used to generate *sna*^{MS2}, *sna*$^{\Delta ATG}$*/CyO-Hb>lacZ*, and *Sna*^{Llama} CRISPR alleles.

Supplementary Data 4: Enhancer sequences for *snail*^{Distal} transgenes, related to Figure 5.

Supplementary Data 5: Single molecule fluorescent *in situ* hybridization probes for endogenous *sna*.

Supplementary Data 6: qPCR primers related to Supplementary Figure 9.

Supplementary Data 7: Simulation results from modelling analysis related to Figure 6.

Supplemental Text: Equivalence of sequential and non-sequential three state transcriptional bursting models.

Models

The live transcription imaging data discriminates models via the distribution of waiting times w separating successive transcription initiation events they produce. Let us suppose that the parametric fit of this distribution selects a sum of three exponentials:

$$S(t) = \mathbb{P}[w > t] = A_1 \exp(\lambda_1 t) + A_2 \exp(\lambda_2 t) + A_3 \exp(\lambda_3 t). \quad (1)$$

In this case BurstDeconv provides five independent parameters $A_1, A_2, \lambda_1 < 0, \lambda_2 < 0, \lambda_3 < 0$ (because $A_1 + A_2 + A_3 = 1$) and concludes that the transcriptional bursting model has three states.

However, several equivalent three states models lead to exactly the same distribution (1) and therefore fit the data equally well. Two such alternative models are indicated in the Figure ST1. These differ by the topology of transitions between the states. In the sequential model, the active state ON is not directly accessible from OFF1; the system must first transition through OFF2 to reach ON. In the non-sequential model, the ON state is directly accessible from both OFF1 and OFF2.

Model Equivalence

In the BurstDeconv pipeline [1] the only parameters that are fitted are $A_1, A_2, \lambda_1, \lambda_2, \lambda_3$, which are common for all three states models. The transition rate parameters of individual three state models are obtained from $A_1, A_2, \lambda_1, \lambda_2, \lambda_3$ by analytical formulas [3, 1, 2]. These formulas are provided below.

The transition rate parameters for the sequential model are given by

$$\begin{aligned} k_{\text{ini}} &= -S_1, \\ k_2 &= \frac{L_1 S_1 S_2 - L_2 S_1^2 + L_3 S_1 - S_2^2}{S_1^3 - S_1 S_2}, \end{aligned}$$

$$
\begin{aligned}
k_4 &= \frac{S_1^2 - S_2}{S_1}, \\
k_1 &= \frac{-1}{(-S_1^2\, S_2^2 + S_2^3 + (S_1^3\, S_2 - S_1\, S_2^2)\, L_1 + (-S_1^4 + S_1^2\, S_2)\, L_2 + (S_1^3 - S_1\, S_2)\, L_3)} \\
&\quad (L_1^2\, S_1^3\, S_2 + L_2^2\, S_1^3 + S_1\, S_2^3 + L_3^2\, S_1 + (-2\, S_1^2\, S_2^2 + (-S_1^4 - S_1^2\, S_2)\, L_2 + (S_1^3 + S_1\, S_2)\, L_3)\, L_1 \\
&\quad + (S_1^3\, S_2 - 2\, L_3\, S_1^2 + S_1\, S_2^2)\, L_2 + (S_1^4 - 3\, S_1^2\, S_2)\, L_3), \\
k_3 &= \frac{(S_1^2 - S_2)\, L_3}{(L_1\, S_1\, S_2 - L_2\, S_1^2 + L_3\, S_1 - S_2^2)}, \qquad (2)
\end{aligned}
$$

and the transition rate parameters for the non-sequential model are given by

$$
\begin{aligned}
k_{\mathrm{ini}} &= -S_1, \\
k_2 &= -\frac{L_1\, S_1 - S_2 \pm \sqrt{L_1^2\, S_1^2 - 2\, L_1\, S_1\, S_2 - 4\, L_3\, S_1 + S_2^2}}{2\, S_1}, \\
k_4 &= \frac{L_1\, S_1^2 + S_1^2\, k_2 - L_2\, S_1 - S_1\, S_2 - S_2\, k_2 + L_3}{2\, k_2\, S_1 + L_1\, S_1 - S_2}, \\
k_1 &= -\frac{k_2\, S_1 + L_1\, S_1 - S_2}{S_1}, \\
k_3 &= -\frac{-S_1^3\, k_2 + L_1\, S_1\, S_2 - L_2\, S_1^2 + S_1\, S_2\, k_2 + L_3\, S_1 - S_2^2}{S_1\, (2\, k_2\, S_1 + L_1\, S_1 - S_2)}, \qquad (3)
\end{aligned}
$$

where

$$
\begin{aligned}
L_1 &= \lambda_1 + \lambda_2 + \lambda_3, \\
L_2 &= \lambda_1\lambda_2 + \lambda_1\lambda_3 + \lambda_2\lambda_3, \\
L_3 &= \lambda_1\lambda_2\lambda_3, \\
S_1 &= A_1\lambda_1 + A_2\lambda_2 + A_3\lambda_3, \\
S_2 &= A_1\lambda_1^2 + A_2\lambda_2^2 + A_3\lambda_3^2. \qquad (4)
\end{aligned}
$$

From the transition rate parameters one can compute the lifetimes T_{OFF1}, T_{OFF2}, T_{ON} and the steady state occupation probabilities P_{OFF1}, P_{OFF2}, P_{ON} of the three states (see Table ST1).

The numerical values of the parameters are given in the Table ST2 for two *sna* genotypes. For both genotypes, the lifetimes of the two OFF states are well separated: T_{OFF1} is one order of magnitude larger than T_{OFF2}. In such a situation, the parameters of the sequential and non-sequential models that fit the data equally well are very similar. In particular, in both descriptions, the effect of the DistalAlt mutation is a modification of the long OFF state lifetime. Indeed, when OFF2 is much shorter than OFF1, it does not matter much whether transitions from OFF1 to ON are direct or occur via the very short intermediate OFF2.

Model	T_{OFF2}	T_{OFF1}^{-1}	T_{ON}^{-1}	P_{ON}	P_{OFF2}	P_{OFF1}
sequential	$k_1 + k_2$	k_3	k_4	$\frac{k_2k_3}{k_1k_4+k_2k_3+k_3k_4}$	$\frac{k_3k_4}{k_1k_4+k_2k_3+k_3k_4}$	$\frac{k_1k_4}{k_1k_4+k_2k_3+k_3k_4}$
non-sequential	k_2	k_1	$(k_3 + k_4$	$\frac{k_1k_2}{k_1k_2+k_1k_4+k_2k_3}$	$\frac{k_1k_4}{k_1k_2+k_1k_4+k_2k_3}$	$\frac{k_2k_3}{k_1k_2+k_1k_4+k_2k_3}$

Table ST1: Reciprocal lifetimes and steady state occupation probabilities for the sequential and non-sequential models.

Snail Genotype	P_{ON} nonseq	P_{ON} seq	T_{ON} nonseq	T_{ON} seq	P_{OFF1} nonseq	P_{OFF1} seq	T_{OFF1} nonseq	T_{OFF1} seq	P_{OFF2} nonseq	P_{OFF2} seq	T_{OFF2} nonseq	T_{OFF2} seq	k_{ini} nonseq	k_{ini} seq
DistalWT	0.34	0.34	43.5	43.5	0.25	0.30	605	640	0.41	0.36	49.35	49.13	0.17	0.17
DistalAlt	0.53	0.53	61.7	61.7	0.07	0.099	265.7	275.3	0.4	0.37	44.6	44.34	0.17	0.17

Table ST2: Parameter values for sequential and non-sequential models that fit data equally well. Units of time are in seconds.

References

[1] Maria Douaihy, Rachel Topno, Mounia Lagha, Edouard Bertrand, and Ovidiu Radulescu. BurstDECONV: a signal deconvolution method to uncover mechanisms of transcriptional bursting in live cells. *Nucleic Acids Research*, 51(16):e88–e88, 2023.

[2] Ovidiu Radulescu, Dima Grigoriev, Matthias Seiss, Maria Douaihy, Mounia Lagha, and Edouard Bertrand. Identifying markov chain models from time-to-event data: an algebraic approach. *Bulletin of Mathematical Biology*, 87(11):1–46, 2025.

[3] Katjana Tantale, Encar Garcia-Oliver, Marie-Cécile Robert, Adèle L'Hostis, Yueyuxiao Yang, Nikolay Tsanov, Rachel Topno, Thierry Gostan, Alja Kozulic-Pirher, Meenakshi Basu-Shrivastava, Kamalika Mukherjee, Vera Slaninova, Jean-Christophe Andrau, Florian Mueller, Eugenia Basyuk, Ovidiu Radulescu, and Edouard Bertrand. Stochastic pausing at latent HIV-1 promoters generates transcriptional bursting. *Nature Communications*, 12(1):4503, Jul 2021.

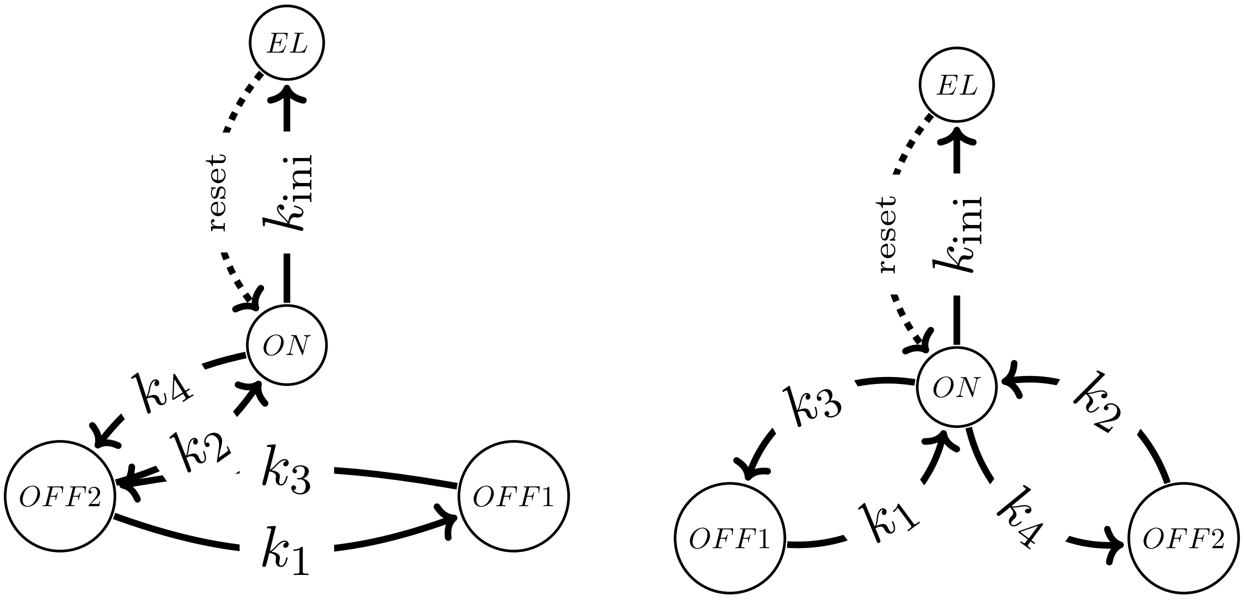

Sequential model **Non-sequential model**

Figure ST1: Two alternative transcriptional bursting models. These two models have the same number of states (three) and can generate exactly the same phase-type distribution and therefore are both compatible with live transcription imaging data. ON is the transcriptionally active state, while OFF1 and OFF2 are transcriptionally inactive states. EL represents the elongating Pol II. Once Pol II initiates elongation, the promoter quickly resets and is ready to initiate transcription again (this reset occurs via a very fast transition). BurstDeconv [1] determines sets of parameters for the two models simultaneously, using analytic formulas to pass from one set of parameters to the other. Some of these parameters are rigorously identical for the best fit models of the two kinds, namely P_{ON}, T_{ON}, k_{ini}. Other parameters are approximately equivalent when the lifetimes of the two OFF states are well separated which is the case here; T_{OFF1} is one order of magnitude larger than T_{OFF2}, see Table ST2.
