# Supplementary material for: Coordinated active repression operates via transcription factor cooperativity and multiple inactive promoter states in a developing organism
Source: Nat Commun. 2025 Sep 1;16:8157. doi: 10.1038/s41467-025-62907-3 (PMC12402238; doi:10.1038/s41467-025-62907-3)
Supplement: Supplementary file 2 — Description of Additional Supplementary Files [file 41467_2025_62907_MOESM2_ESM.pdf]

Description of Additional Supplementary Files

Supplementary Movie 1: Description: Live imaging of snaMS2 representative of nc13-14 beginning at mitosis. Nuclei are detected using His2Av-mRFP and MS2 using MCP-GFP.

Supplementary Movie 2: Description: Live imaging of sogMS2 representative of NC14 beginning at mitosis. Nuclei are detected using His2Av-mRFP and MS2 using MCP-GFP.

Supplementary Movie 3: Description: Live imaging of snaLlama representative of nc13 and nc14 beginning at mitosis. Nuclei are detected using His2Av-mRFP.

Supplementary Movie 4: Description: live imaging of snaDistal -24xMS2-y representative of nc14 beginning at mitosis. Nuclei are detected using His2Av-mRFP and MS2 using MCP-GFP.

Supplementary Movie 5: Description: live imaging of snaDistalAlt -24xMS2-y representative of nc14 beginning at mitosis. Nuclei are detected using His2Av-mRFP and MS2 using MCP-GFP.

Supplementary Movie 6: Description: live imaging of snaDistalMut -24xMS2-y representative of nc14 beginning at mitosis. Nuclei are detected using His2Av-mRFP and MS2 using MCP-GFP.

Supplementary Movie 7: Description: live imaging of snaDistalCore -24xMS2-y representative of nc14 beginning at mitosis. Nuclei are detected using His2Av-mRFP and MS2 using MCP-GFP.
