# Supplementary material for: Coordinated active repression operates via transcription factor cooperativity and multiple inactive promoter states in a developing organism
Source: Nat Commun. 2025 Sep 1;16:8157. doi: 10.1038/s41467-025-62907-3 (PMC12402238; doi:10.1038/s41467-025-62907-3)
Supplement: Supplementary file 10 — Reporting Summary [file 41467_2025_62907_MOESM10_ESM.pdf]

nature portfolio

Corresponding author(s): LAGHA, Mounia
RADULESCU, Ovidiu

Last updated by author(s): Jul 13, 2025

# Reporting Summary

Nature Portfolio wishes to improve the reproducibility of the work that we publish. This form provides structure for consistency and transparency in reporting. For further information on Nature Portfolio policies, see our Editorial Policies and the Editorial Policy Checklist.

## Statistics

For all statistical analyses, confirm that the following items are present in the figure legend, table legend, main text, or Methods section.

| n/a | Confirmed | |
|---|---|---|
| ☐ | ☒ | The exact sample size (*n*) for each experimental group/condition, given as a discrete number and unit of measurement |
| ☐ | ☒ | A statement on whether measurements were taken from distinct samples or whether the same sample was measured repeatedly |
| ☐ | ☒ | The statistical test(s) used AND whether they are one- or two-sided<br>*Only common tests should be described solely by name; describe more complex techniques in the Methods section.* |
| ☒ | ☐ | A description of all covariates tested |
| ☐ | ☒ | A description of any assumptions or corrections, such as tests of normality and adjustment for multiple comparisons |
| ☐ | ☒ | A full description of the statistical parameters including central tendency (e.g. means) or other basic estimates (e.g. regression coefficient) AND variation (e.g. standard deviation) or associated estimates of uncertainty (e.g. confidence intervals) |
| ☐ | ☒ | For null hypothesis testing, the test statistic (e.g. *F*, *t*, *r*) with confidence intervals, effect sizes, degrees of freedom and *P* value noted<br>*Give P values as exact values whenever suitable.* |
| ☐ | ☒ | For Bayesian analysis, information on the choice of priors and Markov chain Monte Carlo settings |
| ☐ | ☒ | For hierarchical and complex designs, identification of the appropriate level for tests and full reporting of outcomes |
| ☒ | ☐ | Estimates of effect sizes (e.g. Cohen's *d*, Pearson's *r*), indicating how they were calculated |

*Our web collection on statistics for biologists contains articles on many of the points above.*

## Software and code

Policy information about availability of computer code

| | |
|---|---|
| Data collection | Data collection was performed using Zen Black v3.2 (Zeiss). |
| Data analysis | BurstDECONV source code is available through GitHub at https://github.com/oradules/BurstDECONV, and also on Zenodo at https://zenodo.org/record/7443044. SegmentTrack is available via GitHub at https://github.com/ant-trullo/SegmentTrack_v4.0) and the post-processing tool developed for this study at https://github.com/ant-trullo/SpotsFiltersTool. NucleiTracker3D is available via GitHub at https://github.com/ant-trullo/NucleiTracker3D. The BCPD algorithm is available via GitHub at https://github.com/mariadouaihy/BCPD_inhomogeneous_transcriptional_signal. Analysis was also performed using Prism 10 (Graphpad) and Microsoft Excel. |

For manuscripts utilizing custom algorithms or software that are central to the research but not yet described in published literature, software must be made available to editors and reviewers. We strongly encourage code deposition in a community repository (e.g. GitHub). See the Nature Portfolio guidelines for submitting code & software for further information.

## Data

Policy information about availability of data

All manuscripts must include a data availability statement. This statement should provide the following information, where applicable:

- Accession codes, unique identifiers, or web links for publicly available datasets
- A description of any restrictions on data availability
- For clinical datasets or third party data, please ensure that the statement adheres to our policy

Data is available upon request from the corresponding authors.

## Research involving human participants, their data, or biological material

Policy information about studies with human participants or human data. See also policy information about sex, gender (identity/presentation), and sexual orientation and race, ethnicity and racism.

| | |
|---|---|
| Reporting on sex and gender | n/a |
| Reporting on race, ethnicity, or other socially relevant groupings | n/a |
| Population characteristics | n/a |
| Recruitment | n/a |
| Ethics oversight | n/a |

Note that full information on the approval of the study protocol must also be provided in the manuscript.

# Field-specific reporting

Please select the one below that is the best fit for your research. If you are not sure, read the appropriate sections before making your selection.

☒ Life sciences ☐ Behavioural & social sciences ☐ Ecological, evolutionary & environmental sciences

For a reference copy of the document with all sections, see nature.com/documents/nr-reporting-summary-flat.pdf

# Life sciences study design

All studies must disclose on these points even when the disclosure is negative.

| | |
|---|---|
| Sample size | For each genotype, a minimum of 2 movies were considered, with each containing between 50-100 imaged nuclei. For each analysis the sample size (number of activated nuclei) is indicated. For nonparametric estimation of the waiting time distribution we have typically used 200 transcription sites resulting in 20 000-50 000 waiting time sample size. The resulting confidence intervals were small enough to allow discrimination between models with 2 states and 3 states at the parametric estimation step of our mathematical modeling. The sample size is large enough to allow robust statistical analysis. |
| Data exclusions | n/a |
| Replication | Multiple embryos were imaged for each condition as stated in the applicable figure legends. |
| Randomization | n/a |
| Blinding | n/a, analysis is performed in a semi-supervised automated manner. |

# Reporting for specific materials, systems and methods

We require information from authors about some types of materials, experimental systems and methods used in many studies. Here, indicate whether each material, system or method listed is relevant to your study. If you are not sure if a list item applies to your research, read the appropriate section before selecting a response.

## Materials & experimental systems

| n/a | Involved in the study | |
|---|---|---|
| ☐ | ☒ | Antibodies |
| ☒ | ☐ | Eukaryotic cell lines |
| ☒ | ☐ | Palaeontology and archaeology |
| ☐ | ☒ | Animals and other organisms |
| ☒ | ☐ | Clinical data |
| ☒ | ☐ | Dual use research of concern |
| ☒ | ☐ | Plants |

## Methods

| n/a | Involved in the study | |
|---|---|---|
| ☒ | ☐ | ChIP-seq |
| ☒ | ☐ | Flow cytometry |
| ☒ | ☐ | MRI-based neuroimaging |

# Antibodies

| | |
|---|---|
| Antibodies used | rabbit anti-Snail (Pimmett, McGehee et al, Development 2025), donkey anti-rabbit AF488 (Life Technologies) |
| Validation | rabbit anti-Snail previously published, validation documents for donkey anti-rabbit AF488 available from manufacturer |

# Animals and other research organisms

Policy information about studies involving animals; ARRIVE guidelines recommended for reporting animal research, and Sex and Gender in Research

| | |
|---|---|
| Laboratory animals | D.melanogaster laboratory strains, as listed in supplementary Data 2 |
| Wild animals | n/a |
| Reporting on sex | n/a, sex of embryos is unknown |
| Field-collected samples | n/a |
| Ethics oversight | n/a |

Note that full information on the approval of the study protocol must also be provided in the manuscript.

# Plants

| | |
|---|---|
| Seed stocks | n/a |
| Novel plant genotypes | n/a |
| Authentication | n/a |
