# Supplementary material for: Coordinated active repression operates via transcription factor cooperativity and multiple inactive promoter states in a developing organism
Source: Nat Commun. 2025 Sep 1;16:8157. doi: 10.1038/s41467-025-62907-3 (PMC12402238; doi:10.1038/s41467-025-62907-3)
Supplement: Supplementary file 12 — Supplementary Data [file 41467_2025_62907_MOESM12_ESM.pdf]

Supplementary Data 1: Kinetic parameters for promoters derived from deconvolution and multi-exponential regression fitting of live imaging data. Minimum and maximum values indicate the boundaries of the error interval. State durations are calculated from the provided switching rates (ki +/-) and time durations for each state are provided as 'T(state)'. State probability values are indicated as 'p(state)'. Bold indicates the most parsimonious appropriate fitting of the data. The table also provides the objective functions and one-sided Kolmogorov-Smirnov test results.

Supplementary Data 2: Drosophila lines used in this manuscript.

Supplementary Data 3: guide RNA and ssODN sequences used to generate snaMS2 , snaΔATG/CyO-Hb>lacZ, and SnaLlama CRISPR alleles.

Supplementary Data 4: Enhancer sequences for snailDistal transgenes, related to Figure 5.

Supplementary Data 5: Single molecule fluorescent in situ hybridization probes for endogenous sna.

Supplementary Data 6: qPCR primers related to Supplementary Figure 9.

Supplementary Data 7: Simulation results from modelling analysis related to Figure 6.
