# Supplementary material for: Coordinated active repression operates via transcription factor cooperativity and multiple inactive promoter states in a developing organism
Source: Nat Commun. 2025 Sep 1;16:8157. doi: 10.1038/s41467-025-62907-3 (PMC12402238; doi:10.1038/s41467-025-62907-3)
Supplement: Supplementary file 13 — Supplementary Data 1-7 [file 41467_2025_62907_MOESM13_ESM.pdf]

Supplementary Data 1: Kinetic parameters for indicated genotypes

Genotype	2 States Model									
	k2+	k2-	k3	T(OFF) (s)	T(On) (s)	Pol II Initiaiton (s/event)	p(OFF)	p(ON)	Objective function	Kolmogorov-Smirnov
***sna* MS2 - active phase (nc13)**	**0.011**	**0.007**	**0.350**	**91**	**142**	**2.85**	**0.392**	**0.608**	**0.095432177**	**0.985401194**
Minimum	0.011	0.005	0.330	91	192	3.03	0.161	0.608		
Maximum	0.027	0.007	0.350	37	142	2.85	0.392	0.839		

Genotype	3 States Model													
	k1+	k1-	k2+	k2-	k3	T(OFF1) (s)	T(OFF2) (s)	T(On) (s)	Pol II Initiaiton (s/event)	p(OFF1)	p(OFF2)	p(ON)	Objective function	Kolmogorov-Smirnov
**snaMS2 - repressed phase (nc14)**	**0.001**	**0.007**	**0.045**	**0.112**	**0.271**	**956**	**22**	**8**	**3.68**	**0.671**	**0.236**	**0.094**	**0.002011144**	**0.961125388**
Minimum	0.001	0.007	0.045	0.112	0.271	956	22	8	3.68	0.184	0.236	0.094		
Maximum	0.010	0.008	0.049	0.116	0.284	100	20	8	3.52	0.671	0.573	0.243		

Genotype	3 States Model													
	k1+	k1-	k2+	k2-	k3	T(OFF1) (s)	T(OFF2) (s)	T(On) (s)	Pol II Initiaiton (s/event)	p(OFF1)	p(OFF2)	p(ON)	Objective function	Kolmogorov-Smirnov
snailDistal	**0.002**	**0.001**	**0.020**	**0.022**	**0.171**	**640**	**49**	**44**	**5.84**	**0.299**	**0.361**	**0.340**	**0.005894326**	**0.059164976**
Minimum	0.002	0.000	0.020	0.022	0.171	237	49	44	5.75	0.029	0.361	0.340		
Maximum	0.004	0.001	0.020	0.022	0.174	640	49	44	5.84	0.299	0.510	0.462		
snailDistalAlt	**0.003**	**0.001**	**0.019**	**0.018**	**0.160**	**342**	**52**	**53**	**6.23**	**0.089**	**0.443**	**0.468**	**0.008572441**	**0.137155036**
Minimum	0.003	0.000	0.019	0.018	0.160	241	46	52	6.08	0.042	0.443	0.468		
Maximum	0.004	0.001	0.022	0.019	0.165	342	52	53	6.23	0.089	0.446	0.512		

Genotype	2 States Model									
	k2+	k2-	k3	T(OFF) (s)	T(On) (s)	Pol II Initiaiton (s/event)	p(OFF)	p(ON)	Objective function	Kolmogorov-Smirnov
snailDistalMut	**0.013**	**0.007**	**0.135**	**78**	**145**	**7.40**	**0.351**	**0.649**	**0.040272118**	**0.507765922**
Minimum	0.013	0.006	0.134	68	145	7.40	0.298	0.649		
Maximum	0.015	0.007	0.135	78	159	7.45	0.351	0.702		
snailDistalCore	**0.028**	**0.006**	**0.124**	**36**	**154**	**8.10**	**0.188**	**0.812**	**0.028555408**	**0.1666541**
Minimum	0.028	0.006	0.124	36	154	8.10	0.188	0.812		
Maximum	0.028	0.006	0.124	36	154	8.10	0.188	0.812		

Supplementary Data 2: Fly lines associated with Pimmett et al. (2025)

Line	In Text Reference	Reference
; ; nos> MCP-eGFP, His2A-mRFP		Gift from T.Fukaya
; *Mat-alpha* :GAL4/CyO; *nos* :GAL4, *nos* >MCP-eGFP, His2A-RFP/*nos* :GAL4, nos>MCP-eGFP, His2A-RFP		Lagha lab
; *snailMS2-3xP3-dsRed/snailMS2-3xP3-dsRed ;*	*snaMS2*	This paper
; *sna* Δ*ATG /* CyO, *hb* >lacZ ;	*snaΔATG*	This paper
sogMS2/sogMS2 ; ;	*sogMS2*	Whitney et al., Development 2022
; *snailLlama/snailLlama* ;	*SnailLlama*	This paper
yw; P{w[+mC] = EGFP-STOP-bcd} ;	*bcd>* GFP	Bothma et al., Cell 2018
w ; *P{w[+mC]=His2Av-mRFP}/ CyO ;*	His2A-RFP	
w1118 ; *P{GD9782}v20876* ;	Paf1 RNAi-A	VDRC 20876
; ; *P{KK100080}VIE-260B*	Paf1 RNAi-B	VDRC 108826
; ; *P{UASp-CycT.H}*	UAS:CycT	Hunt et al., Genome Biology 2024
; ; *P{y[+t7.7] v[+t1.8]=TRiP.HMS00686}attP2*	Nelf-A RNAi	BDSC 32897
; *PBac{sna-MS2-y}* ;	*snaWT* BAC	Bothma et al., eLife 2015
; *PBac{snaΔprimary-MS2-y}* ;	*snaΔPROX* BAC	Bothma et al., eLife 2015
; *PBac{snaΔshadow-MS2-y}* ;	*snaΔDIST* BAC	Bothma et al., eLife 2015
; ; PBPhi(*snaDistal-24xMS2-y)* (VK33)	*snaDistal*	Dufourt et al., Nature Communications 2018
; ; PBPhi(*snaDistalAlt-24xMS2-y)* (VK33)	*snaDistalAlt*	This paper
; ; PBPhi(*snaDistalMut-24xMS2-y)* (VK33)	*snaDistalMut*	This paper
; ; PBPhi(*snaDistalCore-24xMS2-y)* (VK33)	*snaDistalCore*	Ferraro et al., Current Biology 2016

Supplementary Data 3: guide RNA sequences for generation of CRISPR alleles

Target	Sequence
snail – MS2	CGACATATGAATCCCTTAGCAGG
snail – Llama	CGACATATGAATCCCTTAGCAGG, CCCCATGAACGAAGAGTACTAGG
snail – ΔATG (guide)	GTAGTGACCCATTGAATTCGTGG
snail – ΔATG (ssODN)<br>**guide sequence**<br>mutations<br>EcoRI site for screening	TCGATCAGTACCGGAAACTAAAACTTAATCACACACACATCAAAAATGGCCGCC<br>AACTACAAAAGCTGCCCGCTAAA**GTAGTGACCCATTGAATTCGTGG**AGGAGC<br>GTCTGCCACAAACGGAGGCCTTGGCCCTGACCAAGGACTCACAGTTTGCCCA<br>GGATCAGCCGCAGGATCTATCCCTGAAACGGGGTCGCGACGA
White - coffee	ATACCATTCCTGCTCTTTGG

Supplementary Data 4: *snail* Distal Enhancer sequences

Genotype	Sequence
snailDistal (WT)	cgcattgaggtgttttgttggtcaacgccaaacatgtgcttgccacatgccccgtttcggacccattttcaataccaaactcaagcctgtccaggcgaaggtgtgaactatttggcctggtgccttatctacacttagaaaaatattg<br>acataaaatggatttttgaacggtgttaaacaaaacggtgtttgttttaaacaaaatacttcaaaattcgtaaaatacgattatcgttaaaacccatcgtaaccatttaaatatacccttcataccatttacatacttaattattta<br>aaatagaagaggttcaacatatattttccgttttccaaactagttgacattttttgcggtgtaactgtgatagctgtgtaccttcaaagtcccatgtgccagtctctctcaccttggtcctaccttcgacttcgctgggctctccgttttc<br>ccatgagggagttctcctggcccgctcacatatgtcggaactccaactgcggagctgggaaaacaaacaggactggcggtactggttacacatgtgtgagctggccggaccggagcacgtactatgcgcgtaggtgcgactcc<br>ttggattccccagttctccagttcccacacatcctggccgggccacatgatgatgttgctgtgcttttcccgtagcgccaggcgtgtccaaatgttttgttgcggaattcctcaaggcgaggaatctcgcacacattacgagtagtcg<br>gcgctcgcggaaaaaaaacacacgagccaaagttacatatgttctttgggccatttattcaagaaatttccatgtcggccgaaaagggatttccttgtaggcggcgttttcccacgattttataactgggctcgacttcccatcttc<br>ccaccgtccatgtgttgctgggaaatcggcgttgcctttggtttttgccttgcgggggaaaagttctgatttgagtgcgctcgagactttccagctaggagcaggacatgtggccggtagccagtgccactcagtacctcactcag<br>tctaatggccagaacaccgcggtcatctcgagtggacaggttgtaaagtggccacacccgccgaccggacacttgactctatgcgccggcgcagcatcgcaggtagtcatctggaacaggtaaacatttgccaatatctcacgc<br>tgacctatgacacgtgtcaggtgtctttgtcgtggattcgggcaagtgtgaaacaacctactacctgttgaggcaataatgattatgaatacgaatgaattaacacggagaacgataagagggccaattgcatttgttccttcttc<br>caaaacgagctgcaaacggcagctaattgtattttaataattataaatccgtgttaaattgtttgaaatttaataaattggttcctttgcaaaaacagcttaaatggaaatactgcaattttgtccatattttattgtcatcgaattttt<br>caaatgg
snailDistalAlt	cgcattgaggtgttttgttggtcaacgccaaacatgtgcttgccacatgccccgtttcggacccattttcaataccaaactcaagcctgtccaggcgaaggtgtgaactatttggcctggtgccttatctacacttagaaaaatattg<br>acataaaatggatttttgaacggtgttaaacaaaacggtgtttgttttaaacaaaatacttcaaaattcgtaaaatacgattatcgttaaaacccatcgtaaccatttaaatatacccttcataccatttacatacttaattattta<br>aaatagaagaggttcaacatatattttccgttttccaaactagttgacattttttgcggtgtaactgtgatagctgtgtaccttcaaagtcccatgtgccagtctctctcaccttggtcctaccttcgacttcgctgggctctccgttttc<br>ccatgagggagttctcctggcccgctcacatatgtcggaactccaactgcggagctgggaaaacaaacaggactggcggtactggttacacatgtgtgagctggccggaccggagcacgtactatgcgcgtaggtgcgactcc<br>ttggattccccagttctccagttcccacacatcctggccgggccacatgatgatgttgctgtgcttttcccgtagcgccaggcgtgtccaaatgttttgttgcggaattcctcaaggcgaggaatctcgcacacattacgagtagtcg<br>gcgctcgcggaaaaaaaacacacgagccaaagttacatatgttctttgggccatttattcaagaaatttccatgtcggccgaaaagggatttccttgtaggcggcgttttcccacgattttataactgggctcgacttcccatcttc<br>ccaccgtccatgtgttgctgggaaatcggcgttgcctttggtttttgccttgcgggggaaaagttctgatttgagtgcgctcgagactttccagctaggagcagcgtaagtgcccggtagccagtgccactcagtacctcactcag<br>tctaatggccagaacaccgcggtcatctcgagtggacaggttgtaaagtggcccgtaatagtgaccggacacttgactctatgcgccggcgcagcatcgcaggtagtcatctgcgtaatagtcgtatttgccaatatctcacgct<br>gacctatgacacgtgtcaggtgtctttgtcgtggattcgcgtaagtgcgaaacaacctactacctgttgaggcaataatgattatgaatacgaatgaattaacacggagaacgataagagggccaagcgtaatagtcgtttcttc<br>caaaacgagctgcaaacggcagctaattgtattttaataattataaatccgtgttaaattgtttgaaatttaataaattggttcctttgcaaaaacagcttaaatggaaatactgcaattttgtccatattttattgtcatcgaattttt<br>caaatgg
snailDistalMut	cgcattgaggtgttttgttggtcaacgccaaacatgtgcttgccacatgccccgtttcggacccattttcaataccaaactcaagcctgtccaggcgaaggtgtgaactatttggcctggtgccttatctacacttagaaaaatattg<br>acataaaatggatttttgaacggtgttaaacaaaacggtgtttgttttaaacaaaatacttcaaaattcgtaaaatacgattatcgttaaaacccatcgtaaccatttaaatatacccttcataccatttacatacttaattattta<br>aaatagaagaggttcaacatatattttccgttttccaaactagttgacattttttgcggtgtaactgtgatagctgtgtaccttcaaagtcccatgtgccagtctctctcaccttggtcctaccttcgacttcgctgggctctccgttttc<br>ccatgagggagttctcctggcccgctcacatatgtcggaactccaactgcggagctgggaaaacaaacaggactggcggtactggttacacatgtgtgagctggccggaccggagcacgtactatgcgcgtaggtgcgactcc<br>ttggattccccagttctccagttcccacacatcctggccgggccacatgatgatgttgctgtgcttttcccgtagcgccaggcgtgtccaaatgttttgttgcggaattcctcaaggcgaggaatctcgcacacattacgagtagtcg<br>gcgctcgcggaaaaaaaacacacgagccaaagttacatatgttctttgggccatttattcaagaaatttccatgtcggccgaaaagggatttccttgtaggcggcgttttcccacgattttataactgggctcgacttcccatcttc<br>ccaccgtccatgtgttgctgggaaatcggcgttgcctttggtttttgccttgcgggggaaaagttctgatttgagtgcgctcgagactttccagctaggagcagcgtaagtgcccggtagccagtgccactcagtacctcactcag<br>tctaatggccagaacaccgcggtcatctcgagtggacaggttgtaaagtggcccgtaatagtgaccggacacttgactctatgcgccggcgcagcatcgcaggtagtcatctgcgtaatagtcgtatttgccaatatctcacgct<br>gacctatgacacgtgtcaggtgtctttgtcgtggattcgcgtaagtgcgaaacaacctactacctgttgaggcaataatgattatgaatacgaatgaattaacacggagaacgataagagggccaagcgtaatagtcgtttcttc<br>caaaacgagctgcaaacggcagctaattgtattttaataattataaatccgtgttaaattgtttgaaatttaataaattggttcctttgcaaaaacagcttaaatggaaatactgcaattttgtccatattttattgtcatcgaattttt<br>caaatgg
snailDistalCore (Pimmett, Dejean et al., 2021)	ccttggtcctaccttcgacttcgctgggctctccgttttcccatgagggagttctcctggcccgctcacatatgtcggaactccaactgcggagctgggaaaacaaacaggactggcggtactggttacacatgtgtgagctggcc<br>ggaccggagcacgtactatgcgcgtaggtgcgactccttggattccccagttctccagttcccacacatcctggccgggccacatgatgatgttgctgtgcttttcccgtagcgccaggcgtgtccaaatgttttgttgcggaattcc<br>tcaaggcgaggaatctcgcacacattacgagtagtcggcgctcgcggaaaaaaaacacacgagccaaagttacatatgttctttgggccatttattcaagaaatttccatgtcggccgaaaagggatttccttgtaggcggcgtt<br>ttcccacgattttataactgggctcgacttcccatcttcccaccgtccatgtgttgctgggaaatcggcgttgcctttgg

Supplementary Data 5: single molecule FISH probes

snail endogenous smFISH probes

Probe	Sequence	Probe Type
snail_1	tctcaacgagagctgaggtg	smFISH
snail_2	gagtatagagcggtggttgc	smFISH
snail_3	tgggtaaatcgggagatcgg	smFISH
snail_4	agttttagtttccggtactg	smFISH
snail_5	ccatttttgatgtgtgtgtg	smFISH
snail_6	ttagcgggcagcttttgtag	smFISH
snail_7	ctcctccacgaagacaatgg	smFISH
snail_8	caaactgtgagtccttggtc	smFISH
snail_9	cgtttcagggatagatcctg	smFISH
snail_10	tgctgataatcctgggtctc	smFISH
snail_11	acatagtcacgtttcggttc	smFISH
snail_12	ccggtgtttttgaaaggttc	smFISH
snail_13	agttggagctagagctggag	smFISH
snail_14	tagtccacgcatatggattt	smFISH
snail_15	gattaatcgtggtgggggtg	smFISH
snail_16	atcacaaaggcggactggaa	smFISH
snail_17	cagagatcggattgcaaccg	smFISH
snail_18	atctgctggtagctgtagac	smFISH
snail_19	aaccggtttccagatcggat	smFISH
snail_20	actgaaagatcctctggctc	smFISH
snail_21	cggcagtgggatgtcatttc	smFISH
snail_22	gcctcatcgaaaaggtggaa	smFISH
snail_23	tgtaggagtatcccgatgag	smFISH
snail_24	catgattggcggcaacactc	smFISH
snail_25	gcacttgaagcggtagtttt	smFISH
snail_26	atcgaggtggagtacatctt	smFISH
snail_27	aactgacggtgcttggacag	smFISH
snail_28	ttcttctcctgattacactc	smFISH
snail_29	aatggtggtgtacagctttc	smFISH
snail_30	gtgcggatgtgcatcttcag	smFISH
snail_31	caaatggggcacttgcaggg	smFISH
snail_32	agggtcgagagaaggccttg	smFISH
snail_33	aaaggcttctctccagtgtg	smFISH
snail_34	caaaggatcgtgggcagtcg	smFISH
snail_35	gatgagctcgcaggttcgag	smFISH
snail_36	tacttcttgacgtccacgtg	smFISH
snail_37	gaaagatttgtggcacacct	smFISH
snail_38	tgcttgttcaggagcgacat	smFISH
snail_39	tagtgatggtgcagttggag	smFISH
snail_40	atatgtcgagaatcctacgc	smFISH
snail_41	taattgtgtcctgctaaggg	smFISH
snail_42	gcggaatgtgagtttgctta	smFISH
snail_43	attgtctgtttgtttggtct	smFISH
snail_44	gcaccaaaaccgaatcgact	smFISH
snail_45	atgctgcgtgtgacaatgag	smFISH
snail_46	acagttggcttaacagtact	smFISH
snail_47	ttcttctctttaagctagga	smFISH

MS2 smiFISH probes

Probe	Sequence	Probe Type
MS2_1	GATCGTCGTCGTTTGAAGATTCGACCTGG	smiFISH
MS2_2	CGGCTGATGCTCGTGCTTTCTTGGCA	smiFISH
MS2_3	CGTAGGATCTGATGAACCCTGGAATACTGG	smiFISH

yellow smFISH probes

Probe	Sequence	Probe Type
yellow_1	atcagggtcacaagaatcca	smFISH
yellow_2	actatatcgctcctgaagtt	smFISH
yellow_3	tttagtcgggtattcgggaa	smFISH
yellow_4	tataatctccactagccaga	smFISH
yellow_5	ttcgactccaacaggtagag	smFISH
yellow_6	gtgacgaataaccgattgcc	smFISH
yellow_7	aaactgcggtccatgtttat	smFISH
yellow_8	gccaatctggatacggaatt	smFISH
yellow_9	caatctccagctgtatttga	smFISH
yellow_10	gtaggcagtggtaatactgt	smFISH
yellow_11	ccacactcatccactttaat	smFISH
yellow_12	cacggattagtggtggtatt	smFISH
yellow_13	gtatccgtggtcaagtcaaa	smFISH
yellow_14	tagctcgtatctccgaattc	smFISH
yellow_15	gtatttggatttgtgtccac	smFISH
yellow_16	cacggcaatgttagctatga	smFISH
yellow_17	atcatcgcaatttttgccta	smFISH
yellow_18	tatcccaattcatcggcaaa	smFISH
yellow_19	cccaggagtaagcaatcaag	smFISH
yellow_20	agaatctccaggacttgttc	smFISH
yellow_21	cctcaatggatcggggaaaa	smFISH
yellow_22	cccattggaagttaatacca	smFISH
yellow_23	ataccaaatataccctcctc	smFISH
yellow_24	cgatcgaatgggcgaaaggg	smFISH
yellow_25	agtacagggtacgataacca	smFISH
yellow_26	cgatgacttgctaacggact	smFISH
yellow_27	aaaatcctcgtggatacggc	smFISH
yellow_28	catgatagctatcttccgtc	smFISH
yellow_29	ccgttcatctaaggcaacaa	smFISH
yellow_30	cacgtgaagtggtatgggag	smFISH
yellow_31	acagctcaattccatcatcg	smFISH
yellow_32	gagtacggcattgatgagtg	smFISH
yellow_33	ccacaatgccatgaaattgc	smFISH
yellow_34	ttttcacatcggccggaaaa	smFISH
yellow_35	acccaaacgtttttgttctc	smFISH
yellow_36	gcaagaaaacgggcatccta	smFISH
yellow_37	aagggagccgtgtaaattcg	smFISH
yellow_38	aggcgttattcctcaaatca	smFISH
yellow_39	acggcttgttttggtattga	smFISH
yellow_40	atataacggtggacccattg	smFISH
yellow_41	tttctgtggcaagacaggac	smFISH
yellow_42	cgggcaaataagtgcgactt	smFISH
yellow_43	tggagactacattgcctgaa	smFISH
yellow_44	ggacccacagaatttgtaga	smFISH
yellow_45	ccgttgtgctggttgaaaat	smFISH
yellow_46	gaccacttgtctcgtaattt	smFISH
yellow_47	gggttgatgggtgggaaata	smFISH
yellow_48	aaccttgatgctgatgatgc	smFISH

Supplementary Data 6: qPCR Primers

Name	Sequence
paf1_F	CACCGCTTCGTGCAGTACAA
paf1_R	CCAAATCGTGTTCCGTCAGC
CycT_F	CCGGCCCGTCTGAAGTCTA
CycT_R	CCTTGCTGTTAGCTGTCCGAT
Rpl13_F	AGCGGCATGTGAAGACCTG
Rpl13_R	AAGACGGCCTTAGCCTTCTTG

Supplementary Data 7: Parameters for kinetic modelling of repression

**Symbol**	**Value**	**Justification**
k_{ini}	0.35 s^{-1}	nc13 and nc14 last segment value
$(k_2^m)_{act}$	0.007 s^{-1}	nc13 value
$(k_2^p)_{act}$	0.011 s^{-1}	nc13 value
$(k_1^p)_{rep}$	0.001 s^{-1}	nc14 last segment value
$(k_1^m)_{rep}$	0.0075 s^{-1}	nc14 last segment value
$(k_2^m)_{rep}$	0.11 s^{-1}	nc14 last segment value
$(k_2^p)_{rep}$	0.045 s^{-1}	nc14 last segment value
$(k_1^p)_{act}$	0.067 s^{-1}	free parameter, fitted
nh	13	free parameter, fitted
th	5.95	free parameter, fitted
