# Supplementary material for: Coordinated active repression operates via transcription factor cooperativity and multiple inactive promoter states in a developing organism
Source: Nat Commun. 2025 Sep 1;16:8157. doi: 10.1038/s41467-025-62907-3 (PMC12402238; doi:10.1038/s41467-025-62907-3)
Supplement: Supplementary file 11 — Transparent Peer Review file [file 41467_2025_62907_MOESM11_ESM.pdf]

nature portfolio

Peer Review File

Coordinated active repression operates via transcription factor cooperativity and multiple inactive promoter states in a developing organism

Corresponding Author: Dr Mounia Lagha

This manuscript has been previously reviewed at another journal. This document only contains information relating to versions considered at Nature Communications.

This file contains all reviewer reports in order by version, followed by all author rebuttals in order by version.

Version 0:

Reviewer comments:

Reviewer #1

(Remarks to the Author)
The authors have now fully addressed my comments and I support publication.

(Remarks on code availability)

Open Access This Peer Review File is licensed under a Creative Commons Attribution 4.0 International License, which permits use, sharing, adaptation, distribution and reproduction in any medium or format, as long as you give appropriate credit to the original author(s) and the source, provide a link to the Creative Commons license, and indicate if changes were made.

In cases where reviewers are anonymous, credit should be given to 'Anonymous Referee' and the source.
The images or other third party material in this Peer Review File are included in the article's Creative Commons license, unless indicated otherwise in a credit line to the material. If material is not included in the article's Creative Commons license and your intended use is not permitted by statutory regulation or exceeds the permitted use, you will need to obtain permission directly from the copyright holder.
To view a copy of this license, visit https://creativecommons.org/licenses/by/4.0/
